# Adaptations of gummivorous primate gut-associated bifidobacteria to type-II arabinogalactan utilization

**DOI:** 10.1093/ismejo/wrag210

**Published:** 2026-08-19

**Authors:** Yuki Sasaki, Tomoya Kozakai, Moe Inoue, Mikiyasu Sakanaka, Toshihiko Katoh, Hiroki Kaneko, Hiroo Imai, Toshitaka Odamaki, Kiyotaka Fujita, Takane Katayama

**Affiliations:** Graduate School of Biostudies, Kyoto University, Kyoto, 606-8502, Japan; Graduate School of Biostudies, Kyoto University, Kyoto, 606-8502, Japan; Graduate School of Biostudies, Kyoto University, Kyoto, 606-8502, Japan; Graduate School of Biostudies, Kyoto University, Kyoto, 606-8502, Japan; Graduate School of Biostudies, Kyoto University, Kyoto, 606-8502, Japan; Biotics Research Institute, R & D Division, Morinaga Milk Industry Co., Ltd, Zama, Kanagawa, 252-8583, Japan; Institute for the Evolutionary Origins of Human Behavior, Kyoto University, Inuyama, Aichi, 484-8506, Japan; Graduate School of Biostudies, Kyoto University, Kyoto, 606-8502, Japan; Biotics Research Institute, R & D Division, Morinaga Milk Industry Co., Ltd, Zama, Kanagawa, 252-8583, Japan; Faculty of Agriculture, Kagoshima University, Kagoshima, 890-0065, Japan; Graduate School of Biostudies, Kyoto University, Kyoto, 606-8502, Japan

**Keywords:** gummivorous primates, type-II arabinogalactan, bifidobacteria, dietary adaptation

## Abstract

Bifidobacteria inhabiting the primate gut exhibit host-dependent genetic diversification, particularly in their gene repertoire related to carbohydrate metabolism, suggesting adaptation to host diets. However, these diverse genetic traits remain poorly associated with specific dietary components. Here, through enzymatic and genetic analyses, we demonstrate that several *Bifidobacterium* species residing in the gut of gummivorous primates, such as marmosets, possess previously uncharacterized pathways for the efficient utilization of type-II arabinogalactan (AG), the major polysaccharide component of tree gums. The assimilation pathways comprises two key components: a bifunctional β-1,3-galactanase that cleaves the AG backbone via both endo- and exo-mode actions and/or an ATP-binding cassette transporter that internalizes the released arabinogalactan oligosaccharides (AGOs) into cells. Data mining of deposited metagenomic datasets suggested that the endo/exo-β-1,3-galactanase and the AGO transporter contribute to cross-feeding interactions within *Bifidobacterium* communities in the gut of gummivorous primates. Our study not only highlights molecular strategies employed by certain *Bifidobacterium* species to adapt to the dietary habits of a host but may also inform probiotic intervention strategies for the health and welfare of these primates in captive settings.

## Graphical abstract

**Graphical Abstract:**
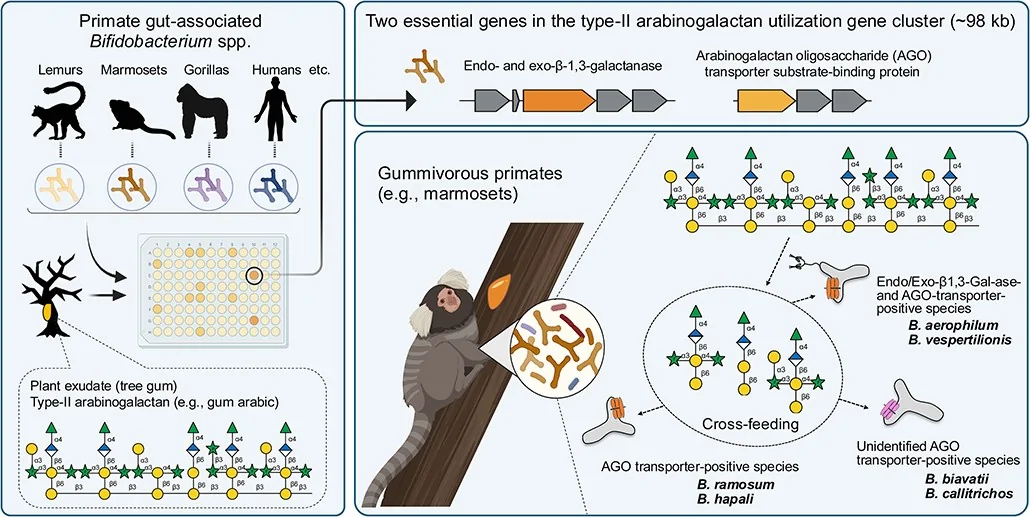

## Introduction

Gut microbes not only exhibit co-phylogeny with their hosts but also show genomic diversification at species and strain levels, as revealed by comparative genomic analyses [1–3]. Co-phylogeny and diversification are considered to be associated with host genetic and ecological differences that persist across generations [4, 5]. Under multifaceted long-time pressures imposed by hosts, certain gut microbes that eventually gained specific metabolic and/or ecological adaptations to their hosts could have been retained in the gut microbial community. Such adaptations are represented by the presence of gene sets required for detoxification of harmful compounds [6], production of vitamins [7], beneficial immunomodulatory compounds [8], and degradation of host-inaccessible nutrients in the gut [6, 9, 10]. As such, the genus *Bifidobacterium* has recently attracted considerable attention as a model microbe for studying co-evolutionary trajectories with its hosts. The majority of *Bifidobacterium* species have been isolated from the gastrointestinal tract of animals including mammals, birds, and insects, although some are derived from sewage and fermented milk [11]. A recent pangenomic analysis unveiled the diversified genetic traits in the genus, particularly in the repertoires of carbohydrate-active enzymes (CAZymes), which potentially reflect species-specific adaptation to host and/or dietary glycans present in the gut niche [3, 12, 13]. The most intensely studied case is the adaptation to human milk oligosaccharides (HMOs) [14]. The gene sets responsible for the assimilation of HMOs are enriched in infant gut-associated *Bifidobacterium* species and less prevalent among species inhabiting the gut of human adults and non-human primates [2, 15, 16]. However, apart from this well-recognized co-evolutionary relationship, our knowledge linking specific genetic traits in bifidobacteria to host- or diet-derived glycans remains largely limited to predictions based on *in silico* analyses [3, 12, 17].

Members of the family Callitrichidae (e.g. the genera *Callithrix, Saguinus*, and *Leontopithecus*) are small-bodied New World monkeys native to South America that consume plant exudates such as tree gums. In particular, marmosets devote ~30%–70% of their foraging time to gum feeding [18]. Potentially reflecting this unique dietary habit (gummivory), their gut microbiota differs from those of other New World monkeys that primarily consume fruits (frugivory) and tree leaves (folivory) [17]. Specifically, whereas non-gummivorous primates harbour no or low levels of bifidobacteria in their gut microbiota, gummivorous primates harbour these taxa at a relative abundance of 10%–80% of the total gut microbiota. In addition, the predominance of bifidobacteria in the gut of gummivorous primates persists throughout adulthood [17, 19], although the abundance is reported to vary among individuals and to change based on environmental contexts (e.g. wild vs. captive populations). Plant gums are water-soluble, viscous exudates secreted from beneath the tree bark and contain complex glycans known as type-II arabinogalactans (AGs) as major constituents. Type-II AGs are high-molecular-weight polysaccharides (240–580 kDa) consisting of a β-1,3-galactan backbone highly substituted with galactose (Gal) with a β-(1 → 6)-linkage. The branched Gal moieties are further modified by l-arabinose (Ara), Gal, glucuronic acid (GlcA), 4-*O*-methyl-GlcA, and/or l-rhamnose (Rha), resulting in the formation of structurally diverse side chains depending on the plant origins. Commercially available gum derived from *Acacia senegal* (Fabaceae, the legume family), known as gum arabic, is one of the most structurally characterized AGs [20] and has been used as a model substrate to examine gut microbial utilization mechanisms. Previous studies have focused on two prominent human gut microbial species, *Bifidobacterium longum* subsp. *longum* (*B. longum*) [21] and *Bacteroides thetaiotaomicron* [22], which revealed overlapping yet distinct gum arabic utilization pathways between two representative Gram-positive and Gram-negative microbes (Supplementary Fig. S1). *Bifidobacterium longum* first releases disaccharides α-d-Gal-(1 → 3)-l-Ara and β-l-Ara*p*-(1 → 3)-l-Ara from the side chains using 3-*O*-α-d-galactosyl-α-l-arabinofuranosidase (GAfase), a glycoside hydrolase family 39 (GH39) enzyme [23]. This removal enables α-l-arabinofuranosidase (ArafE), harbouring both GH43 subfamily 22 (GH43_22) and GH43_34 domains, to trim α-(1 → 3)- and α-(1 → 4)-linked Ara moieties from the side chains, respectively [21]. This trimming subsequently allows exo-β-1,3-galactanase (Exo-β1,3-Gal-ase, GH43_24) to act on the β-1,3-galactan backbone, producing α-l-Rha-(1 → 4)-β-d-GlcA-(1 → 6)-β-d-Gal-(1 → 6)-Gal tetrasaccharide (termed oligosaccharide S4) and β-1,6-galactobiose (Supplementary Fig. S1A) [21]. *Bifidobacterium longum* can utilize only α-d-Gal-(1 → 3)-l-Ara, Ara, and β-1,6-galactobiose among the released sugars, leaving β-l-Ara*p*-(1 → 3)-l-Ara and oligosaccharide S4 unconsumed, which is cross-fed to other microbes [21]. Although Exo-β1,3-Gal-ase is highly conserved, both upstream enzymes (GAfase and ArafE) are present in <6.8% of *B. longum* genomes [23], rendering the ability of this subspecies to utilize type-II AG highly strain-dependent. Regarding *Ba. thetaiotaomicron*, its ability to utilize gum arabic AG relies on other microbes that possess an extracellular endo-β-1,3-galactanase (Endo-β1,3-Gal-ase, GH43_24), which partially depolymerizes type-II AG [22] (Supplementary Fig. S1B). The resulting partially degraded products are then transported into the periplasm via a Sus system and hydrolysed into constituent monosaccharides by a concerted action of Endo-β1,3-Gal-ase (GH43_24), Exo-β1,3-Gal-ase (GH43_24), and other CAZymes, followed by internalization into the cytoplasm [22]. The prevalence of genes encoding GH43_24 enzymes is relatively high in the *Actinomycetota* (which includes the genus *Bifidobacterium*) and *Bacteroidota* phyla compared with other phyla [22, 24], and gum arabic AG is reported to have prebiotic effects in certain human populations [25].

Given the relatively high abundance of bifidobacteria in the gut of callitrichids, we hypothesized that *Bifidobacterium* species inhabiting gummivorous primates are genetically more specialized for the utilization of type-II AGs. In this study, we identified previously unknown, highly efficient type-II AG assimilation pathways within *Bifidobacterium* communities in the callitrichid gut. Data mining of deposited datasets suggested that these pathways contribute to cross-feeding interactions within *Bifidobacterium* communities in the gut of these animals. This study adds to the understanding of diet-dependent evolutionary trajectories between gut microbes and their primate hosts, and may also provide a basis for effective probiotic interventions for gummivorous primates, particularly marmosets that are known to be susceptible to gastrointestinal diseases under captive conditions [26].

## Materials and methods

### Chemicals

Gum arabic (*A. senegal*) was purchased from Sigma-Aldrich (St. Louis, MO, USA). To remove mono- and oligosaccharides, the gum was dissolved in water (29%, *w/v*) and precipitated by adding ethanol at a final concentration of 80% in the presence of 1 mM HCl. The precipitate was recovered and extensively dialysed against water. Three oligosaccharide constituents of gum arabic AG (see Fig. 2G): S4 (see above), α-l-Rha-(1 → 4)-β-d-GlcA-(1 → 6)-[α-l-Ara*f*-(1 → 3)-][α-l-Ara*f*-(1 → 4)-]β-d-Gal-(1 → 6)-d-Gal (termed S5-A), and α-l-Rha-(1 → 4)-β-d-GlcA-(1 → 6)-[α-d-Gal-(1 → 3)-α-l-Ara*f*-(1 → 3)-][α-l-Ara*f*-(1 → 4)-]β-d-Gal-(1 → 6)-d-Gal (termed S5-GA) were prepared as described previously [20, 21, 23] and used as authentic standard sugars. All other chemicals were of analytical grade.

### Sugar analysis

*High-performance anion-exchange chromatography with pulsed amperometric detection (HPAEC-PAD*)*─*An ICS-3000 system (Dionex Corp., Sunnyvale, CA, USA) equipped with CarboPac PA-1 column (φ2 × 250 mm) was used for analysing mono- and oligosaccharides. The elution was performed at a flow rate of 0.25 ml/min with the following gradient system between eluent A (0.25 M NaOH) and eluent B (1 M CH_3_COONa) in water: 40% A and 0.5% B for 0–5 min, 40% A and 0.5%–50% B for 5–30 min, and 40% A and 50% B for 30–35 min.

*Thin-layer chromatography (TLC)─*Aliquots (1 μl) of culture supernatants or reaction mixtures were spotted onto silica gel plates (Silica gel 60 aluminium plate, Sigma-Aldrich), and the plates were developed using a solvent system of *n*-butanol/acetic acid/water (2:1:1 by volume). Sugars were visualized with a diphenylamine-aniline-phosphoric acid reagent [27].

*Quantification of reducing sugars─*The amounts of reducing sugars released from gum arabic AG were measured using the bicinchoninic acid (BCA) method or the Somogyi–Nelson method [28].

### Bacterial cultivation

*Bifidobacterium* strains were obtained from RIKEN Bioresource Center (Ibaraki, Japan) and NITE Biological Resource Center (Chiba, Japan) (Supplementary Table S1). For pre-cultivation, de Man, Rogosa, and Sharpe broth supplemented with 0.34% (*w/v*) sodium ascorbate and 0.02% (*w/v*) cysteine-HCl (MRS-CS) was used. Strains were grown overnight at 37°C under anoxic conditions using an AnaeroPack (Mitsubishi Gas Chemical, Tokyo, Japan) or an InvivO_2_ 400 anoxic chamber (Ruskinn Technology, Bridgend, UK) filled with gas consisting of 85% N_2,_ 10% CO_2_, and 5% H_2_. The pre-cultivated cells were harvested by centrifugation, washed twice with sugar-free MRS-CS, and used to inoculate MRS-CS medium, in which glucose (Glc) was replaced with gum arabic AG or *Ba*Endo/Exo-β1,3-Gal-ase (see Results)-digested gum arabic AG (1%), to give an initial optical density at 600 nm (OD_600_) of 0.01 or 0.025. Growth was evaluated by measuring OD_600_ values at the indicated time points. Growth experiments were performed with biological duplicates or triplicates.

### *In silico* genomic analysis of *Bifidobacterium aerophilum* JCM 30941^T^

The genomic sequence of *B. aerophilum* JCM 30941^T^ was retrieved from the NCBI database (GCA_010667685.1) [29]. CAZymes were identified using the dbCAN3 server with HMMER [30], and their cellular localization was predicted by the SignalP algorithm implemented on the server.

### Expression and purification of recombinant proteins

The gene with locus tag GFD25_RS02180 from *B. aerophilum* was amplified by PCR using the primer pair wild-type (WT)-f/r (Supplementary Table S2). The primers were designed to construct a recombinant protein lacking the N-terminal signal peptide and C-terminal transmembrane region, corresponding to amino acid residues 33–1742. The fragment was inserted into the NdeI and XhoI sites of pET23b to generate a C-terminally hexahistidine-tagged protein. An In-Fusion HD Cloning Kit (Takara Bio, Shiga, Japan) was used for ligation. After sequence confirmation, the resulting plasmid pMI062 was introduced into *Escherichia coli* BL21(DE3) Δ*lacZ*/pRARE2 [31]. The transformants were grown in LB medium supplemented with ampicillin (100 μg/ml) and chloramphenicol (7.5 μg/ml) at 18°C, and when the OD_600_ value reached 0.5, isopropyl-β-d-thiogalactopyranoside was added at a final concentration of 0.1 mM. Following additional cultivation at 18°C for 24 h, the cells were harvested by centrifugation and disrupted by ultrasonication in the lysis buffer consisting of 50 mM sodium phosphate and 300 mM NaCl (pH 7.4). After centrifugation, the supernatant was applied onto a column filled with TALON metal-affinity resin (Clontech Laboratories Inc., CA, USA). The recombinant protein was purified according to the manufacturer’s instructions. The purified fraction was desalted and concentrated using an Amicon Ultracel-50 K centrifugal filter unit (Millipore, MA, USA), during which the buffer was exchanged with 20 mM Tris–HCl (pH 8.0). Endo-β1,3-Gal-ase and Exo-β1,3-Gal-ase 1 from *Ba. thetaiotaomicron* were also recombinantly expressed in *E. coli* BL21(DE3) Δ*lacZ*/pRARE2 and purified as described previously [22]. The purity of the proteins was evaluated by sodium dodecyl sulfate-polyacrylamide gel electrophoresis, followed by Coomassie Brilliant Blue staining (Quick-CBB, Wako Pure Chemical, Osaka, Japan). Protein was quantified using the theoretical molar absorption coefficients at 280 nm (M^−1^ cm^−1^): 356 620 for GFD25_RS02180; 117 370 for *Bt*Endo-β1,3-Gal-ase; 119 555 for *Bt*Exo-β1,3-Gal-ase 1.

### Characterization of the protein with locus tag GFD25_RS02180

The optimal reaction conditions for the GFD25_RS02180 protein were determined using gum arabic AG as the substrate (2%). For examining the optimal pH, 50 mM sodium acetate buffer (pH 4.0–6.0), MES buffer (pH 6.0–7.0), and HEPES buffer (pH 7.5–8.0) were used. The optimal temperature was assessed using a 50 mM sodium acetate buffer (pH 5.0) by incubating the reaction mixtures at a temperature ranging between 25°C and 60°C. The reaction was terminated by heating at 100°C for 5 min. Reducing sugars were quantified using the BCA method as described above. Oligosaccharide release from gum arabic AG (2%) in the presence of the WT enzyme (0.63 nM) was monitored over time by TLC and HPAEC-PAD analyses under the optimized conditions. When comparing the gum arabic AG hydrolysis activities of WT and mutant enzymes, all enzymes were added at a final concentration of 20 nM. Recombinant *Bt*Endo-β1,3-Gal-ase and *Bt*Exo-β1,3-Gal-ase 1 were also included in the analysis. The amounts of released reducing sugars were determined using the Somogyi–Nelson method as described above.

### Multiple alignment of GH43_24 domains

Multiple amino acid sequence alignment of the GFD25_RS02180 protein with previously characterized Exo-β1,3-Gal-ases from *Ba. thetaiotaomicron, B. longum, Clostridium thermocellum, Fusarium oxysporum, Irpex lacteus, Phanerodontia chrysosporium, Streptomyces avermitilis, Streptomyces* sp., and *Sphingomonas* sp. and an Endo-β1,3-Gal-ase from *Ba. thetaiotaomicron* [22, 24, 32–38] was performed using the MUSCLE algorithm implemented in MEGA11 [39] and visualized using Jalview 2.11.5.0 [40]. GenBank accessions of these enzymes are listed in Supplementary Information (Supplementary Table S3). GH43_24 domains were identified using InterPro 92.0 [38].

### Site-directed mutagenesis

Double mutations, D588A/Q705A or D1183A/Q1295A, were introduced into the GFD25_RS02180 gene by site-directed mutagenesis with pMI062 as a template. The KOD Plus mutagenesis kit (Toyobo Co., Ltd, Osaka, Japan) was used. Primer pairs used are A-DA-f/r, A-QA-f/r, B-DA-f/r, and B-QA-f/r (Supplementary Table S2). After sequence confirmation of the 0.9-kb SmaI-SacI region containing D588A/Q705A substitutions and the 1.6-kb EcoRI-KpnI region containing D1183A/Q1295A substitutions, these regions were excised and replaced with the corresponding regions of the parental plasmid pMI062, respectively, using LigationMix (Takara Bio). Expression and purification were conducted similarly as described for the WT enzyme.

### Phylogenetic analysis of GH43_24 domains

Phylogenetic analysis of GH43_24 members was performed using 508 non-redundant representative sequences retrieved from the dbCAN database (HMMdb v14) [41]. A total of 3 621 GH43_24-like sequences in the database were first clustered using CD-HIT v4.8.1 at a 70% amino acid sequence identity threshold [42], yielding 836 sequences. These sequences were then combined with 12 experimentally characterized enzymes [22, 24, 32–38] and the GFD25_RS02180 protein analysed in this study. The combined dataset was searched against the dbCAN database using HMMER v3.4 [43], and GH43_24 domain regions were extracted. Sequences shorter than 200 amino acids were removed, except one experimentally characterized enzyme, yielding a final dataset of 508 representative sequences (Supplementary Table S4). Multiple sequence alignment was performed using MAFFT v7.505 [44], and poorly aligned positions and gap-rich columns were removed using trimAl v1.5 with the gap-threshold option (-gt 0.5) [45], resulting in an alignment of 243 amino acid positions. A maximum-likelihood phylogenetic tree was constructed using IQ-TREE v2.0.7 [46] with the best-fit substitution model selected by ModelFinder [47], and branch support assessed using 1000 ultrafast bootstrap [48] and 1000 SH-aLRT replicates [49]. The tree was visualized using iTOL v7 [50].

### Targeted gene deletions in *B. aerophilum*

The scarless gene deletion system using temperature-sensitive plasmid pMSK197 [51] was employed to delete the GFD25_RS02180, GFD25_RS01960, and GFD25_RS02050 genes individually in *B. aerophilum* JCM 30941^T^. Briefly, 1-kb upstream and downstream flanking regions of each target gene were inserted in tandem into pMSK197, and the resulting plasmid was introduced into *B. aerophilum* cells by electroporation [51]. Following the isolation of transformants (first crossover) by incubating the cells on plates containing spectinomycin (Sp) (30 μg/ml) at 40°C, plasmid excision from the genome (second crossover) was induced by incubating the colonies at 30°C. Subsequently, colonies that showed sensitivity to Sp were picked, and genomic rearrangement at the locus was assessed by genomic PCR with primers designed to anneal outside the region used for recombination (Supplementary Table S2). The intended disruption was confirmed by direct sequencing of the PCR products.

### Analysis of arabinogalactan utilization gene conservation within the genus *Bifidobacterium*

Genomic sequences of 98 *Bifidobacterium* strains representing distinct (sub)species were downloaded from the NCBI database to build a local database. Their accession numbers and sequence completeness (%) are listed together with the strain identifiers and isolation sources in Supplementary Information (Supplementary Table S5). The resulting database was queried with the amino acid sequences of the GFD25_RS02180 and GFD25_RS02050 proteins and other CAZymes encoded in the gene cluster in the genome of *B. aerophilum* JCM 30941^T^.

### Complete genome sequencing of *Bifidobacterium vespertilionis* NBRC 113380^T^

The complete genome sequence of *B. vespertilionis* NBRC 113380^T^ was determined by integrating short- and long-read sequencing data. Genomic DNA was extracted using a standard bead-beating method. Short-read sequencing libraries were prepared and sequenced on a DNBSEQ G400 platform (2 × 150 bp) (MGI, Shenzhen, China) as described previously [52]. For long-read sequencing, DNA size selection was performed based on the protocol recommended by the supplier [53] (Nanopore Technologies, Oxford, UK). Libraries were prepared with the Native Barcoding Kit 24 V14 and sequenced on a MinION device with an R10.4.1 flow cell, in accordance with the manufacturer’s instructions. Basecalling was performed using Dorado (v1.1.1) with the dna_r10.4.1_e8.2_400bps_sup@v5.2.0 model to generate FASTQ files. Raw Nanopore reads were trimmed using Chopper v0.5.0 [54] to remove 40 bases from both the 5′ and 3′ ends, and reads shorter than 1000 bp or with an average quality score below 10 were discarded. Subsequently, Filtlong v0.2.1 [55] was applied to retain the top 95% of bases ranked by quality, with enforcing a minimum read length of 1000 bp. Genome assembly was performed using the Autocycler v0.5.2 pipeline [56] following the developer’s instructions. Long-read sequences were divided into four subsets and assembled using multiple assemblers: Flye v2.9.5 [57], Canu v2.3 [58], myloasm v0.2.0 [59], miniasm v0.3 [60], metaMDBG v1.0 [61], necat v0.0.1 [62], NextDenovo v2.5.2 [63], and Raven v1.8.3 [64]. The resulting assemblies were combined to generate consensus sequences via Autocycler. The advantages of using a multi-assembler approach over using a single assembler have already been demonstrated [56]. Consensus sequences were reoriented to start at *dnaA* using Dnaapler v1.3.0 [65]. After assessing the circularity using Bandage v0.9.0 [66], two rounds of polishing were performed on the long-read assemblies using Medaka [67]. Short reads trimmed with fastp v1.0.1 [68] were then used for additional polishing with Polypolish v0.6.0 [69], which was followed by PyPolca v0.4.0 [70] with the “--careful” option enabled. CheckM2 v1.1.0 [71] indicated 100.0% completeness and 1.13% estimated contamination. Annotation was performed using Bakta v1.9.4 [72] and dbCAN3 [30].

### Metagenomic data mining

Twenty-one publicly available metagenomic datasets (PRJNA847605) [73], reported to comprise reads obtained from pooled faecal and anal swab samples collected from 74 golden lion tamarins and 40 marmosets, were downloaded. Relative abundances of bacteria including *Bifidobacterium* at the genus and species levels were determined using MetaPhlAn4 v4.1.1 [74] with the vJun23_202403 database. Four datasets (LrRVf, LrIgarf, LrNEs1f, and MLDRV), obtained from faecal samples collected from 30 golden lion tamarins, contained no reads corresponding to bacteria and were therefore excluded. Consequently, eight datasets comprising reads obtained from 44 golden lion tamarin samples and nine datasets comprising reads obtained from 40 marmoset samples were used for downstream procedures. Low-quality reads and adapter sequences were removed using fastp v0.24.0 with the default settings. Host-derived sequences were removed using Bowtie2 v2.4.1, in accordance with the previous study [73]. Metagenomic reads with a length of ≥31 bp were selected, and paired-end reads with overlapping sequences were merged using BBMerge v38.26 [75]. For read pairs that could not be merged, only one mate was retained for downstream analysis. The resulting datasets contained the mean ± SD of 1 218 100 ± 671 997 read pairs, ranging from 281 471 to 2 506 696, per pooled sample. Seqkit v2.9.0 [76] was used to determine the total number of reads in each sample. A blastn analysis was performed using the nucleotide sequence of *B. vespertilionis* NBRC 113380^T^ as the query, with thresholds set at ≥90% for both sequence identity and coverage. The abundance of valid hits was normalized to 1 million reads per sample. The results of the data mining analysis are summarized in Supplementary Information (Supplementary Table S6).

### Statistical analysis

Statistical analyses were performed with GraphPad Prism 10.4.1 (GraphPad Software, MA, USA). *P*-values <.01 were considered statistically significant.

## Results

### *Bifidobacterium* species with a prominent ability to assimilate type-II AG

Forty-one *Bifidobacterium* strains, representing 40 (sub)species, were assessed for their ability to assimilate gum arabic AG, the most structurally characterized type-II AG. We selected these *Bifidobacterium* (sub)species based on the reported co-phylogeny with their primate hosts and the varied prevalence of CAZymes in their genomes [12]. These strains were isolated from primates belonging to either the Lemuridae, Cebidae, Callitrichidae, or Hominidae family (Supplementary Table S1). Type strains were used for the analysis, except for *Bifidobacterium bifidum*, for which JCM 1254 was used [27, 77]. *Bifidobacterium longum* JCM 7052, a known gum arabic AG degrader [21] (Supplementary Fig. S1A), was included in the analysis as the positive control. We screened strains for gum arabic AG assimilation by subtracting OD_600_ values obtained for sugar-free medium from those obtained for gum arabic AG-supplemented medium (Fig. 1A), because several strains exhibited non-negligible growth in sugar-free medium (Supplementary Fig. S2A). These strains were recently found to possess the gene set required for utilizing ascorbic acid [2], which was added to the medium as a reducing agent. All tested strains except *Bifidobacterium hapali* grew well in Glc-supplemented medium (Supplementary Fig. S2B).

**Figure 1:**
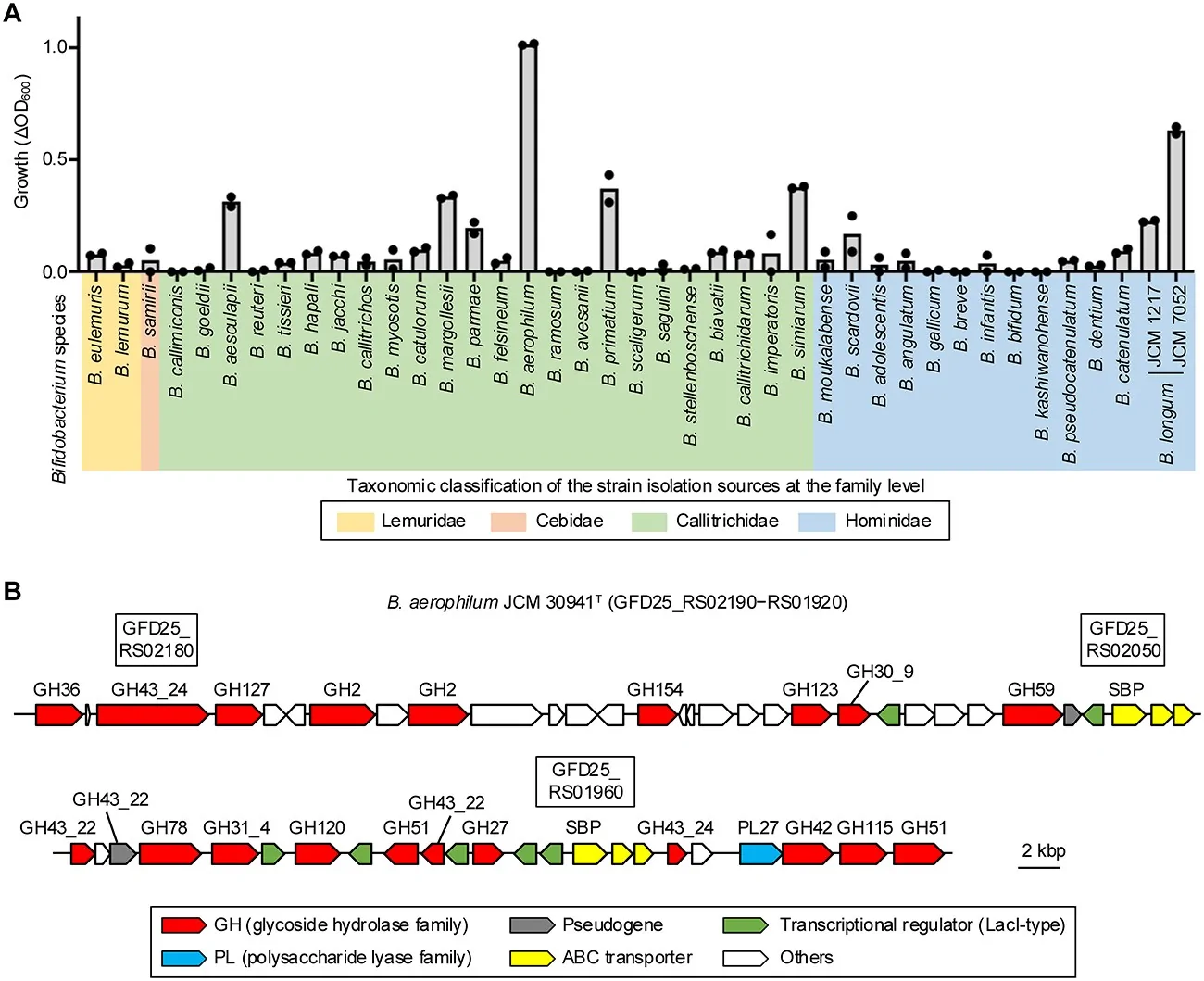
Type-II AG utilization by primate gut-associated *Bifidobacterium* species, and the structure of the gene cluster predicted to be involved in type-II AG utilization in *B. aerophilum*. (A) Growth of *Bifidobacterium* strains in medium supplemented with 1% gum arabic AG. OD_600_ values after 48 h of incubation were corrected by subtracting the values obtained for the same medium without sugar supplementation (ΔOD_600_) (Supplementary Fig. S2A). The species names and their isolation sources (family-level classification of host primates) are indicated. *Bifidobacterium* strain identifiers and their isolation hosts are provided in Supplementary Information (Supplementary Table S1). The type strain of each species was used with the exception of *B. bifidum*, for which JCM 1254 was substituted [27]. Regarding *B. longum*, in addition to the type strain, a known gum arabic AG degrader, JCM 7052 (Supplementary Fig. S1A) [21, 23], was included in the analysis as a positive control. Values obtained from biological duplicates were plotted, with bars representing the mean. (B) Schematic representation of the gene cluster predicted to be involved in type-II AG utilization in the genome of *B. aerophilum* JCM 30941^T^. The diagram was created using drawGeneArrows3 (https://www.ige.tohoku.ac.jp/joho/labhome/tool.html). CAZymes were predicted by dbCAN3 [30] and are shown with their family (subfamily) numbers. Annotations of the other genes are based on the GenBank record. Accession numbers and their predicted functions are provided in Supplementary Information (Supplementary Table S8). The locus tags of the three genes analysed in this study are enclosed by boxes.

The highest growth on gum arabic AG was observed for *B. aerophilum* JCM 30941^T^, an isolate from the cotton-top tamarin (*Saguinus oedipus*) (Fig. 1A). Moderate growth was observed for *Bifidobacterium aesculapii, Bifidobacterium margollesii, Bifidobacterium primatium*, and *Bifidobacterium simiarum*, whose isolation hosts also belong to the Callitrichidae family. The growth of these four strains was less robust than that of *B. longum* JCM 7052. Efficient degradation of gum arabic AG by *B. aerophilum* was evident, based on the results obtained in the TLC analysis of post-cultivation supernatant (Supplementary Fig. S2C). In addition to sugars corresponding to oligosaccharides S4 and S5-GA (see Fig. 2G), a mixture of larger oligosaccharides appeared as a smeared band. *B. aesculapii* produced a spot corresponding to oligosaccharide S4, aligning with our previous study showing that its genome encodes a protein orthologous to Exo-β1,3-Gal-ase from *B. longum* (Supplementary Fig. S1A) [17]. Apparent growth of *B. margollesii, B. primatium*, and *B. simiarum* might result from partial utilization of side chain constituent sugars, because they have extracellular GHs presumably acting on α-l-arabinofuranosyl and β-galactosyl linkages [21, 78]. Taken together, these results indicated that *B. aerophilum* possesses the most efficient enzymatic system to utilize gum arabic AG among primate gut-associated *Bifidobacterium* species examined in this study.

**Figure 2:**
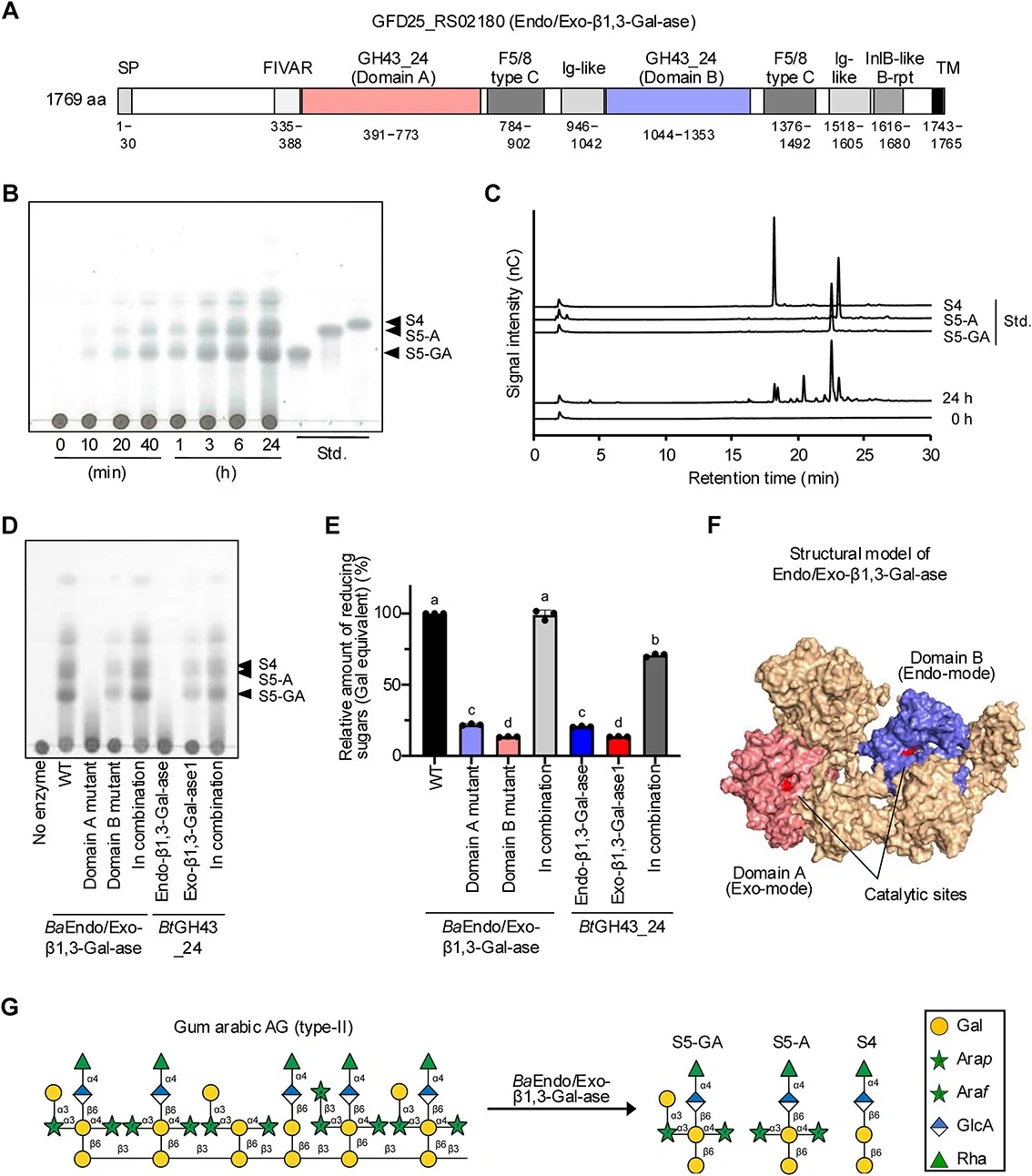
Efficient hydrolysis of type-II AG by the bifunctional endo- and exo-β-1,3-galactanase (Endo/Exo-β1,3-Gal-ase, GFD25_RS02180) from *B. aerophilum*. (A) Domain structure of the protein with locus tag GFD25_RS02180 (Endo/Exo-β1,3-Gal-ase) from *B. aerophilum* JCM 30941^T^. Functional domain names are shown with the amino acid numbering. SP, signal peptide; FIVAR, found in various architectures domain; F5/8 type C, coagulation factor 5/8, C-terminal domain; Ig-like, immunoglobulin-like fold; InlB-like_B-rpt, Internalin B-like, B-repeat; TM, transmembrane. The N-terminal and C-terminal GH43_24 domains, termed Domain A and Domain B, are shown in different colours. (B and C) Time-dependent release of oligosaccharides from gum arabic AG by *Ba*Endo/Exo-β1,3-Gal-ase. Purified recombinant enzyme (0.63 nM, Supplementary Fig. S3A) was incubated with gum arabic AG (2%), and samples collected at the indicated time points were analysed by TLC (B) and HPAEC-PAD (C). Authentic oligosaccharides (S4, S5-A, and S5-GA, see G) [21] were used as standards. Representative data obtained in three independent experiments are presented. (D and E) Dual-mode hydrolysis of gum arabic AG by *Ba*Endo/Exo-β1,3-Gal-ase. Purified WT enzyme, Domain A mutant (D588A/Q705A), Domain B mutant (D1183A/Q1295A), or a combination of the two mutants (each at 20 nM) was incubated with gum arabic AG (1%) for 24 h. For comparison, purified recombinant Endo-β1,3-Gal-ase (BT_0264) and Exo-β1,3-Gal-ase 1 (BT_0265) from *Ba. thetaiotaomicron* ATCC 29148^T^ (Supplementary Figs S1B and S3A) [22] were included. The reaction products were analysed by TLC (D) and HPAEC-PAD (Supplementary Fig. S5). Amounts of reducing sugars (Gal equivalent) in the reaction mixtures, relative to those obtained for WT *Ba*Endo/Exo-β1,3-Gal-ase, are shown after correction by subtracting the values obtained for no-enzyme controls (E). The experiments were performed in duplicate, and representative data are shown (D and E). Reducing sugars were quantified in technical triplicate. Bars and whiskers represent the mean and SD, respectively. Statistical analysis was performed using one-way ANOVA followed by Tukey’s test, with different letters indicating significant differences (*P* < .01). (F) Structural model of *Ba*Endo/Exo-β1,3-Gal-ase generated using AlphaFold2 with default settings [89] was visualized using PyMOL 2.5.0. The same colours as in (A) were used for Domains A and B, with catalytic centres indicated by leader lines. (G) Schematic representation of the hydrolysis of β-1,3-galactan main chain of gum arabic AG by *Ba*Endo/Exo-β1,3-Gal-ase. Representative side chain structures, S5-GA, S5-A, and S4, released by the hydrolysis are shown.

### Potential gene cluster involved in type-II AG utilization by *B. aerophilum*

We searched for CAZymes encoded in the genome of *B. aerophilum* JCM 30941^T^. The dbCAN server [30] predicted the presence of 142 CAZymes including 112 GHs, among which 13 GHs have a signal peptide (Supplementary Table S7). We focused on the GFD25_RS02180 protein that harbours two GH43_24 domains in tandem within a single polypeptide (termed Domain A and Domain B from the N-terminus) (see Fig. 2A), because this GH subfamily comprises Exo-β1,3-Gal-ases and an Endo-β1,3-Gal-ase that hydrolyse the main chain backbone of type-II AG [22, 24, 32–38]. The remaining 12 secretory GHs were classified into GH2, 3, 20, 25, 33, 77, 84, 85, 101, and 146, all of which but GH25 and GH146 include enzymes responsible for the degradation of mammalian glycans [31, 79, 80] and/or plant glycans other than type-II AG. GH25 comprises enzymes that act on bacterial peptidoglycans, whereas GH146 contains β-l-arabinofuranosidase capable of acting on side chains of type-II AG [81]; however, β-Ara*f* residues are scarcely present in gum arabic AG [81]. The GFD25_RS02180 gene is part of a large gene cluster, spanning 98 kb in the genome, together with 20 intracellular GHs, one intracellular polysaccharide lyase (PL), two ATP-binding cassette (ABC) transporters, and seven LacI-type regulators (Fig. 1B). The intracellular CAZyme repertoire contains members of GH2, GH27, GH30_9, GH31_4, GH36, GH42, GH43_22, GH43_24, GH51, GH59, GH78, GH115, GH120, GH123, GH127, GH154, and PL27, some of which exhibited similarity to those of characterized enzymes targeting main and side chains of type-II AG (Supplementary Table S8). Specifically, proteins homologous to GH27 β-l-arabinopyranosidase [82], GH36 α-galactosidase or β-l-arabinopyranosidase [83], GH42 β-galactosidase [84], GH43_22 α-l-arabinofuranosidase [21], GH43_24 exo-β-1,3-galactanase [34], and GH78 α-rhamnosidase [85] were predicted to be required for degrading gum arabic AG.

### The GFD25_RS02180 protein as a bifunctional endo- and exo-β-1,3-galactanase

The GFD25_RS02180 gene encodes a protein of 1769 amino acid residues (aa) with a signal peptide (1–30 aa), uncharacterized found-in-various-architecture (FIVAR) domain (335–388 aa), two GH43_24 domains (Domain A: 391–773 and Domain B: 1044–1353 aa), two coagulation factor 5/8, C-terminal domains (784–902 and 1376–1492 aa), two immunoglobulin-like domains (946–1042 and 1518–1605 aa), an internalin B-like, B-repeat (1616–1680 aa), and a transmembrane region (1743–1765 aa) as functional modules [86] (Fig. 2A). We recombinantly expressed the protein corresponding to 33–1742 aa and, by incubating the purified preparation with gum arabic AG and quantifying reducing sugars released, determined the optimal pH and temperature to be 5.0 and 45–50°C, respectively (Supplementary Fig. S3). Under the optimized reaction conditions, the enzyme released oligosaccharides with their *R*_f_ values identical to those of oligosaccharides S4, S5-A, and S5-GA (see Fig. 2G), as revealed by TLC analysis (Fig. 2B). In this case also, a smeared spot with a lower *Rf* value appeared in a time-dependent manner. Peaks with retention times identical to those of the three authentic oligosaccharides were detected by HPAEC-PAD analysis, demonstrating the digestion of the β-1,3-galactan backbone of gum arabic AG by the enzyme (Fig. 2C). Several other peaks were also detected, indicating the relaxed substrate specificity of this enzyme as well as the structural diversity of gum arabic AG side chains [87]. These findings indicated that the GFD25_RS02180 protein functions as a β-1,3-galactanase.

We wanted to precisely assess the individual functions of the two GH43_24 domains, which share only 26.0% identity, by introducing alanine replacements into the respective putative catalytic residues. The mutated residues were p*K*_a_ modulators Asp^588^ and Asp^1183^ and the catalytic bases Gln^705^ and Gln^1295^ in the respective domains. These residues were predicted by sequence alignment with the structure-determined Exo-β1,3-Gal-ase 2 from *Ba. thetaiotaomicron* (BT_3683, PDB ID: 6EUI) [22, 88] and other previously characterized Endo- and Exo-β1,3-Gal-ases from *Ba. thetaiotaomicron* [22], *B. longum* [24], *C. thermocellum* [32], *F. oxysporum* [35], *I. lacteus* [36], *P. chrysosporium* [37], *S. avermitilis* [33], *Streptomyces* sp. [34], and *Sphingomonas* sp. [38], with sequence identities to Domains A and B ranging from 20.0% to 47.7% (Supplementary Table S3 and Fig. S4). The purified Domain A mutant primarily released larger oligosaccharides from gum arabic AG as a smeared spot, whereas the Domain B mutant primarily released shorter oligosaccharides including S4, S5-A, and S5-GA, as revealed by TLC and HPAEC-PAD analyses (Fig. 2D, Supplementary Fig. S5). The amounts of reducing sugars (Gal equivalent) that were released decreased to 22% and 13% of that of the WT enzyme for Domain A and Domain B mutants, respectively (Fig. 2E). However, the product profiles obtained when the two mutants were used in combination were essentially the same as those obtained for the WT enzyme (Fig. 2D and E and Supplementary Fig. S5). The reducing sugar-producing activity recovered by the combined use of the mutants was higher than the simple sum of the activities that individual mutants exhibited, indicating a synergistic action of two mutant enzymes on the substrate degradation. A similar trend was observed when Endo-β1,3-Gal-ase (BT_0264) and Exo-β1,3-Gal-ase 1 (BT_0265) from *Ba. thetaiotaomicron* [22] were used for the assay individually or in combination. Although the difference in the enzyme source did not affect the mode of action on gum arabic AG (Fig. 2D and Supplementary Fig. S5), the synergistic effect observed for the enzymes from *Ba. thetaiotaomicron* appeared to be lower than that observed for *B. aerophilum* enzymes (Fig. 2E). Although the reason for this difference is unclear, we hypothesize that the affinity of the enzymes to the substrates and/or inhibitory effects of the reaction products may have influenced the synergistic effect. Overall, the results revealed the dual function of GFD25_RS02180 (i.e. exo-β-1,3-galactanase [Domain A] and endo-β-1,3-galactanase [Domain B] activities; hereafter referred to as Endo/Exo-β1,3-Gal-ase) and a potential cooperative action of the two domains during catalysis. A modelled structure generated using AlphaFold2 (ColabFold) [89] suggests the presence of a large cleft between the two catalytic domains (Fig. 2F). This cleft appears to be sufficiently large to accommodate a high-molecular-weight polymer, potentially allowing the predicted catalytic sites of Domain A and Domain B, which face the same side of the enzyme, to act cooperatively and processively on type-II AG. Gal moieties linked with β-(1 → 3)-linkages are generally arranged with their sugar rings oriented in the same direction [90]. Additionally, in type-II AG, side chains are attached primarily to O6 of Gal moieties in the main chain, thereby extending in the direction opposite to the scissile O3-linked glycosidic bond. Cooperative effects on glycoside hydrolysis among multiple catalytic units within a single polypeptide have been reported for other enzymes, including α-l-arabinofuranosidase from *Ruminiclostridium josui* that contains three GH43 modules [91].

Phylogenetic analysis of 508 non-redundant representative GH43_24 members (Supplementary Table S4), including the above-mentioned 12 characterized enzymes and *Ba*Endo/Exo-β1,3-Gal-ase, revealed that Domain A and Domain B are segregated into distinct clades (Supplementary Fig. S6). Domain B of *Ba*Endo/Exo-β1,3-Gal-ase forms a distinct, two-member clade with *Bt*Endo-β1,3-Gal-ase (BT_0264). By contrast, previously characterized Exo-β1,3-Gal-ases and Domain A of *Ba*Endo/Exo-β1,3-Gal-ase are widely distributed throughout the tree, forming distinct clades. The clade containing two Endo-β1,3-Gal-ases (Domain B and *Ba*Endo-β1,3-Gal-ase [BT_0264]) and one Exo-β1,3-Gal-ase (Domain A) branches from the same node as the clade containing three Exo-β1,3-Gal-ases from *C. thermocellum, Sphingomonas* sp., and *S. avermitilis*. These results, together with the widespread prevalence of Exo-β1,3-Gal-ases, suggest that an Endo-β1,3-Gal-ase(s) may have evolved from an Exo-β1,3-Gal-ase(s). However, further enzymatic characterization of ancestral lineages will be necessary to clarify the evolutionary origin of the endo- and exo-mode actions of β-1,3-galactanase. The phylogenetic analysis also revealed that, in addition to *B. aerophilum, Paenibacillus* sp. BIHB 4019, an isolate from the tea rhizoplane, possesses two GH43_24 domains within a single polypeptide. One of the two domains clusters closely with the clade containing Domain B of *Ba*Endo/Exo-β1,3-Gal-ase and *Bt*Endo-β1,3-Gal-ase (BT_0264) and shares >35.8% amino acid sequence identity among them. The other domain falls within the clade containing Domain A of *Ba*Endo/Exo-β1,3-Gal-ase and shares 42.1% sequence identity with Domain A.

### Two essential genes of *B. aerophilum* for gum arabic AG utilization

To examine to what extent the extracellular Endo/Exo-β1,3-Gal-ase contributes to the utilization of gum arabic AG, we deleted the gene in *B. aerophilum*. A double-crossover, scarless deletion was employed to avoid polar effects on the downstream gene (Fig. 1B and Supplementary Fig. S7). The deletion mutant exhibited severely impaired growth on gum arabic AG, compared with the WT strain (Fig. 3A). Whereas oligosaccharides S4, S5-A, and S5-GA and other larger sugars were released and some of these remained unconsumed in the supernatant of the WT strain by the end of incubation (26 h), no oligosaccharides appeared to be released into the supernatant of the deletion mutant over the incubation period (Fig. 3B). The growth of the mutant was comparable to that of the WT strain when gum arabic AG pretreated with purified *Ba*Endo/Exo-β1,3-Gal-ase was used as the carbon source. Under the conditions, spots corresponding to oligosaccharides S4, S5-A, and S5-GA disappeared from the supernatants before the cell growth reached the stationary phase (12 h), regardless of the inoculants. The results not only revealed the indispensable role of Endo/Exo-β1,3-Gal-ase in gum arabic AG utilization by *B. aerophilum* but also indicated the presence of a transporter(s) internalizing arabinogalactan oligosaccharides (AGOs) released by the extracellular Endo/Exo-β1,3-Gal-ase. A transient increase in Rha observed at 12 h of incubation might result from a countermeasure against the rapid increase of intracellular osmotic pressure caused by the hydrolysis of AGOs (up to heptasaccharide) once imported. A similar trend has also been observed for *B. longum* subsp. *infantis* (*B. infantis*) during its growth on HMOs [27]. The organism imports almost all HMOs by ABC transporters and intracellularly degrades them by exo-glycosidases.

**Figure 3:**
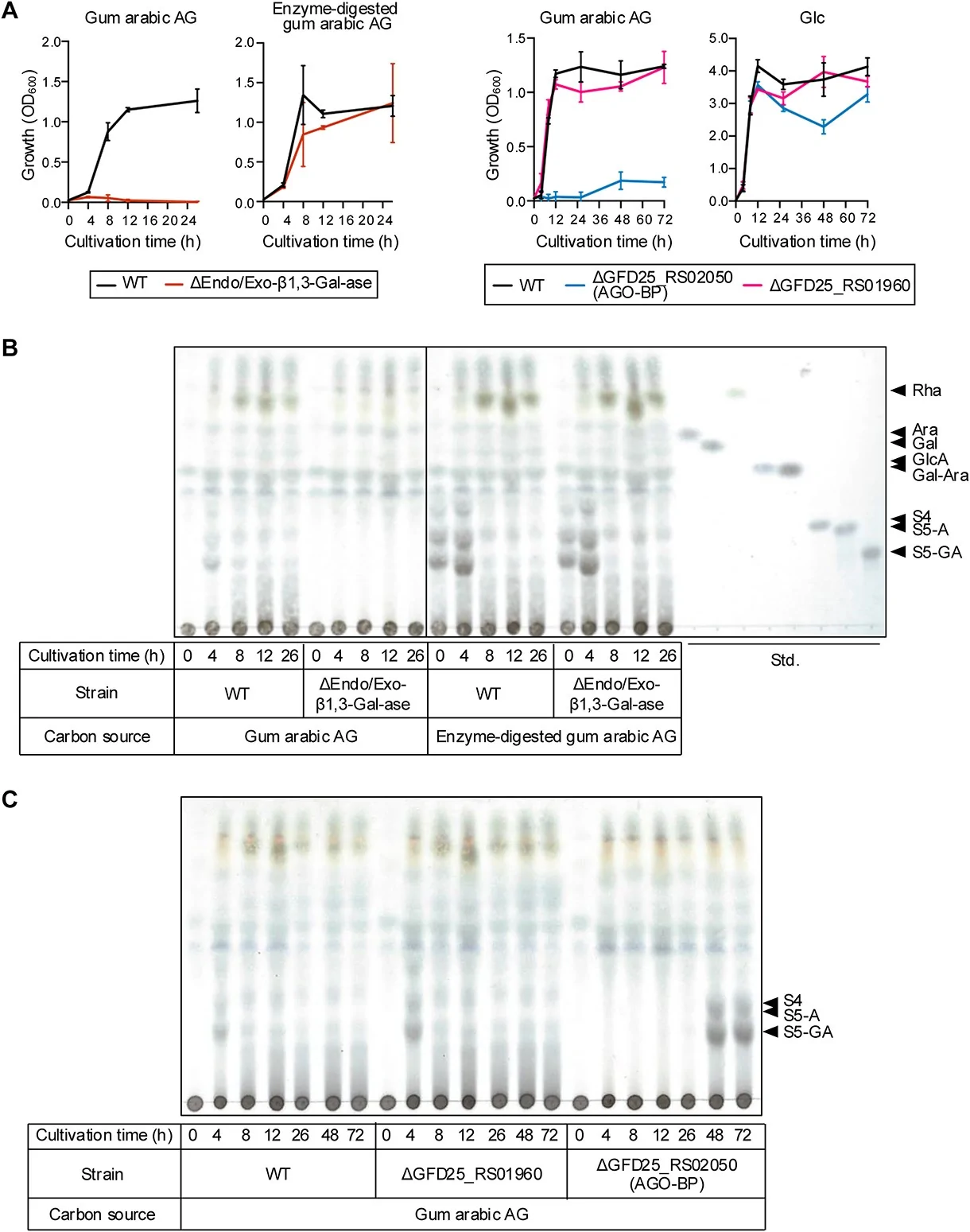
Essential roles of Endo/Exo-β1,3-Gal-ase and AGO-BP in type-II AG assimilation by *B. aerophilum* JCM 30941^T^. (A) Growth of the WT, Δ*Ba*Endo/Exo-β1,3-Gal-ase, ΔGFD25_RS01960, and ΔGFD25_RS02050 (AGO-BP) strains of *B. aerophilum* in the media supplemented with the indicated carbon sources. Proteins with locus tags GFD25_RS01960 and GFD25_RS02050 correspond to SBPs of ABC transporters (Fig. 1B). Gum arabic AG, *Ba*Endo/Exo-β1,3-Gal-ase-digested gum arabic AG (enzyme-digested gum arabic AG), and Glc were used as the sole carbon sources. Samples were taken from the cultures at the indicated time points for measuring OD_600_ values. The lines with whiskers represent the mean ± SD of biological triplicate assays. (B and C) TLC analysis of sugars in the culture medium during incubation. Representative samples collected in (A) were used for the analyses. Culture supernatants of WT and Δ*Ba*Endo/Exo-β1,3-Gal-ase strains grown in the medium supplemented with gum arabic AG or enzyme-treated gum arabic AG were used for the analysis (B). Culture supernatants of WT, ΔGFD25_RS01960, and ΔGFD25_RS02050 (AGO-BP) strains grown on gum arabic AG were used for the analysis (C).

The predicted type-II AG utilization cluster contained two sets of ABC transporter genes, separated by 25 kb (Fig. 1B). Both sets consisted of a solute-binding protein (SBP) gene and two membrane permease genes and lacked an ATPase component gene. This is a trait frequently found in bifidobacterial genomes, which indicates the presence of a universal ATPase for energizing ABC transporters albeit not characterized yet [92]. The two SBPs—GFD25_RS01960 and GFD25_RS02050—shared 48.5% identity in their amino acid sequences. We deleted the two genes individually (Supplementary Fig. S7) and subsequently identified GFD25_RS02050 to be responsible for AGO uptake. Growth of the GFD25_RS01960 gene deletion mutant on gum arabic AG was comparable to that of WT. However, growth of the GFD25_RS02050 gene deletion mutant was severely impaired (Fig. 3A). The TLC profiles of sugars in the supernatants were similar between WT and the GFD25_RS01960 gene deletion mutant (Fig. 3C), whereas accumulation of oligosaccharides including S4, S5-A, and S5-GA was observed at the later stages of incubation in the supernatant of the GFD25_RS02050 gene deletion mutant, indicating the release of the oligosaccharides by the extracellular Endo/Exo-β1,3-Gal-ase without consumption. Despite these phenotypic changes in the growth on gum arabic AG, both mutants grew well on Glc (Fig. 3A). The results demonstrate the essential role of GFD25_RS02050 [hereafter, arabinogalactan oligosaccharide-binding protein (AGO-BP)] for the internalization of AGOs released from gum arabic AG.

### Data mining of publicly available metagenomic datasets

We analysed publicly available shotgun-metagenomic datasets derived from the faeces and anal swabs of gummivorous primates. The datasets contained reads obtained from eight pooled samples, each generated by collecting multiple individual samples of wild or captive golden lion tamarins (*Leontopithecus rosalia*) (*n* = 3–8 per pooled sample, 44 individuals in total) and reads from nine pooled samples generated by collecting multiple individual samples of wild (hybrids of *Callithrix jacchus* and *Callithrix penicillata*) or captive (*C. penicillata*) marmosets (*n* = 2–6 per pooled sample, 40 individuals in total) [73] (Fig. 4A and Supplementary Table S6). The final processed datasets contained a mean ± standard deviation (SD) of 1 218 100 ± 671 997 reads per pooled sample. Species identification of *Bifidobacterium* was performed using the MetaPhlAn4 v4.1.1 [74].

**Figure 4:**
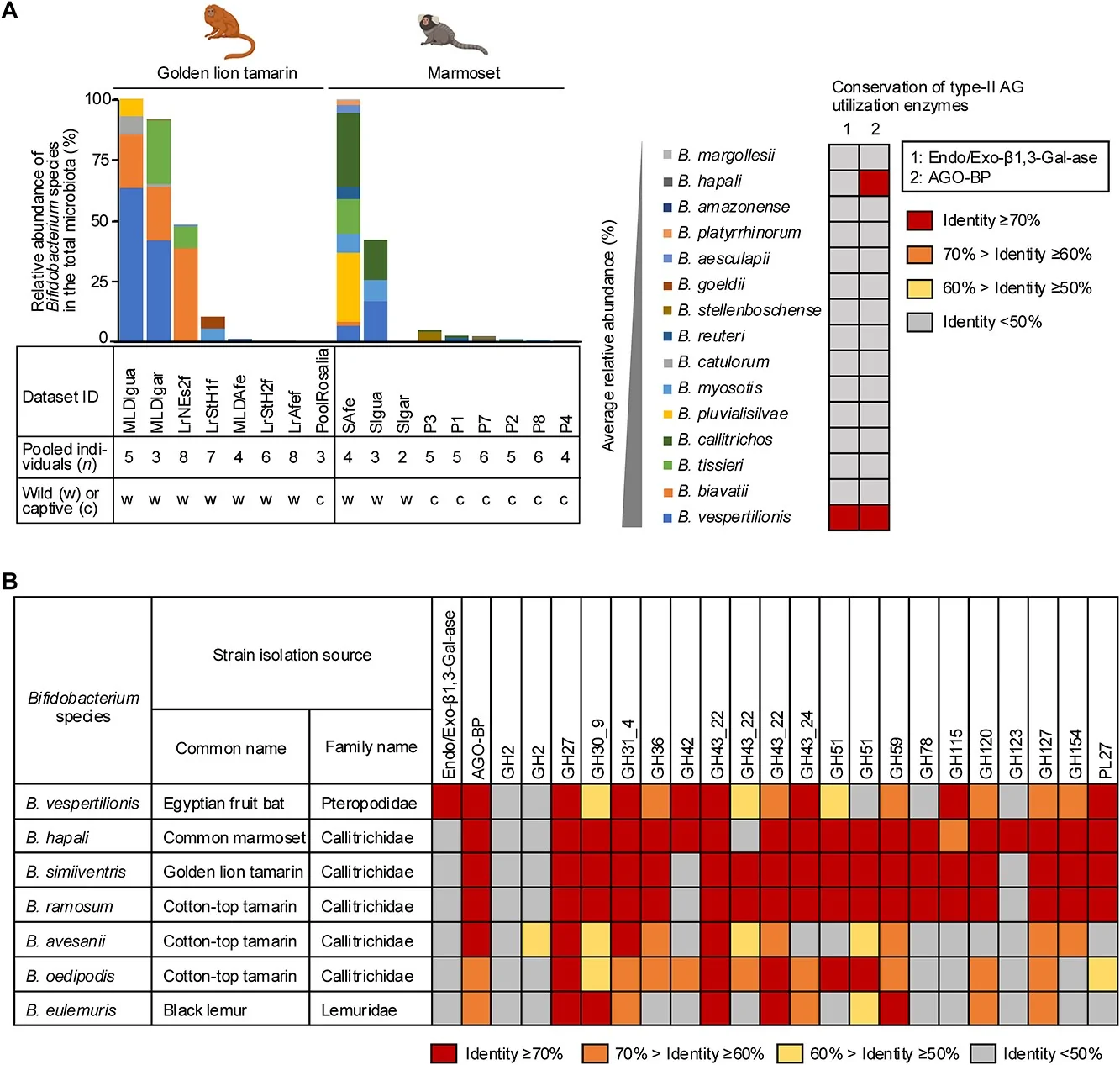
Data mining of deposited faecal metagenome datasets obtained from wild and captive callitrichid monkeys. (A) Relative abundances of *Bifidobacterium* species in the faecal microbiota of wild (w) and captive (c) golden lion tamarins and marmosets, in publicly available metagenomic datasets (PRJNA847605) [73], shown as a stacked bar chart in descending order. The dataset IDs from the original paper are shown below the bar charts. Each of the 17 datasets contains reads obtained from faecal or anal swab samples collected from multiple individuals (*n* = 3–8 for golden lion tamarins and *n* = 2–6 for marmosets). *Bifidobacterium* species assigned by MetaPhlAn4 [74] are listed on the right and ranked according to their average relative abundances (%) in the total microbiota, with different colours representing each species. The occurrence of proteins orthologous to *Ba*Endo/Exo-β1,3-Gal-ase (lane 1) and *Ba*AGO-BP (lane 2) was examined using tblastn searches against the genomes of type strains of the respective *Bifidobacterium* species. The tblastn analyses were performed separately using Domain A (Exo-β1,3-Gal-ase) and Domain B (Endo-β1,3-Gal-ase) sequences of *Ba*Endo/Exo-β1,3-Gal-ase. Sequence identity levels relative to those of *B. aerophilum* are indicated by different colours. (B) Occurrence of proteins orthologous to CAZymes and AGO-BP encoded in the *B. aerophilum* type-II AG utilization cluster (Fig. 1B) within the genus *Bifidobacterium*. Ninety-eight strains with complete or high-quality draft genome sequences, except *Bifidobacterium panos* (Supplementary Table S5), were included in the tblastn analysis, with the sequence coverage threshold set at >70%. The panel shows only the seven *Bifidobacterium* strains that were positive for Endo/Exo-β1,3-Gal-ase and/or AGO-BP. Sequence identity levels relative to those of *B. aerophilum* are indicated by different colours. Results for all 98 strains are listed in Supplementary Information (Supplementary Table S12).

In line with the original report [73], the relative abundance (%) of the genus *Bifidobacterium* in the total microbiota varied considerably across samples, ranging from 0.0% to 100% with an average abundance of 24%. Five out of 17 samples had a bifidobacteria-rich microbiota with their relative abundance exceeding 40% at the genus level. Reads corresponding to 15 *Bifidobacterium* species were detected; however, none of the reads were assigned to *B. aerophilum*. Instead, the highest relative abundance was obtained for *B. vespertilionis* with an average of 7.6%, ranging from 0.0% to 64%. The strain was reportedly isolated from an Egyptian fruit bat (*Rousettus aegyptiacus*). We determined the complete genome sequence of *B. vespertilionis* NBRC 113380^T^ (Supplementary Tables S9–S11) and found that the strain possesses proteins orthologous to *Ba*Endo/Exo-β1,3-Gal-ase and *Ba*AGO-BP with 75.5% and 72.7% identity, respectively. Particularly high identity was detected for Domain A (83.9%) and Domain B (80.5%) regions of the two Endo/Exo-β1,3-Gal-ases. The orthologues encoding Endo/Exo-β1,3-Gal-ase and AGO-BP were found to form a gene cluster spanning 27 kb (termed cluster-1) in the genome of *B. vespertilionis*, together with GH genes predicted to be responsible for the intracellular degradation of side chains of type-II AG (Supplementary Fig. S8A and Supplementary Table S11). Some genes that are present in the type-II AG assimilation cluster of *B. aerophilum* were absent in cluster-1 and located in a distant cluster (termed cluster-2) in the genome. Reflecting this genetic repertoire, *B. vespertilionis* grew well on gum arabic AG to an extent comparable to *B. aerophilum* and released AGOs into the supernatant when the cell suspension was incubated with the gum arabic AG, although the AGO profile on TLC was slightly different from that of *B. aerophilum* (Supplementary Fig. S8B and C). The other 14 species detected in the metagenomic datasets are *Bifidobacterium biavatii, Bifidobacterium tissieri, Bifidobacterium callitrichos, Bifidobacterium pluvialisilvae, Bifidobacterium myosotis, Bifidobacterium catulorum, Bifidobacterium reuteri, Bifidobacterium stellenboschense, Bifidobacterium goeldii, Bifidobacterium aesculapii, Bifidobacterium platyrrhinorum, Bifidobacterium amazonense, Bifidobacterium hapali*, and *Bifidobacterium margollesii*, whose average relative abundance ranged from 5.0% to 0.027% in a decreasing order. The tblastn search showed that none of the type strains of these 14 species encode proteins homologues to Domain A and Domain B regions of *Ba*Endo/Exo-β1,3-Gal-ase with >50.0% identity; however, *B. hapali* was found to possess a protein orthologous to *Ba*AGO-BP with 90.4% identity. An extended genomic survey against 98 *Bifidobacterium* species (Supplementary Table S5), including the above 15 species, revealed that, in addition to *B. vespertilionis* and *B. hapali*, five other species—*Bifidobacterium simiiventris, Bifidobacterium ramosum, Bifidobacterium avesanii, Bifidobacterium oedipodis*, and *Bifidobacterium eulemuris*—possess *Ba*AGO-BP orthologous proteins with ≥64.0% identity. None of the 98 species other than *B. vespertilionis* was positive for Endo/Exo-β1,3-Gal-ase. Genomes of the Endo/Exo-β1,3-Gal-ase- and/or AGO-BP-positive species exhibited varying degrees of conservation of the predicted type-II AG utilization enzymes (Fig. 4B and Supplementary Table S12). Except for *B. eulemuris*, all these species were isolated from the faeces of gummivorous callitrichid monkeys. *B. eulemuris* was reportedly isolated from the black lemur *(Eulemur macaco*), a member of the family Lemuridae (Supplementary Table S1), which also exhibits gummivory, albeit to a limited extent [93].

### Endo/Exo-β1,3-Gal-ase-mediated potential cross-feeding within the *Bifidobacterium* communities in the gut of gummivorous primates

The AGO utilization ability of several *Bifidobacterium* species was assessed using gum arabic AG pretreated with purified *Ba*Endo/Exo-β1,3-Gal-ase. Untreated gum arabic AG was also included in the analysis for reference. The selected strains were the type strains of *B. biavatii, B. tissieri, B. callitrichos, B. myosotis, B. reuteri, B. ramosum*, and *B. hapali*, which were detected in the metagenomic data mining and/or identified as AGO-BP-positive species in the genomic survey as mentioned (Fig. 4 and Supplementary Table S12). *B. vespertilionis* was included in the analysis as the positive control. The results revealed that, in addition to AGO-BP-positive *B. hapali* and *B. ramosum*, two AGO-BP-negative species—*B. biavatii* and *B. callitrichos*—utilize AGOs derived from gum arabic AG (Fig. 5A). Oligosaccharides corresponding to S4, S5-A, and S5-GA disappeared from the supernatants of the four strains after 48 h of incubation, as revealed by the TLC analysis. The genomes of *B. biavatii* JCM 17299^T^ and *B. callitrichos* JCM 17296^T^ appeared to lack extracellular CAZymes that act on AGOs and intact type-II AG (Supplementary Table S13), and consistent with this, both strains showed negligible growth on intact gum arabic AG. These results implied the occurrence of an unidentified AGO transporter(s) in at least these two *Bifidobacterium* species. We examined the above-mentioned metagenomic datasets to see whether Endo/Exo-β1,3-Gal-ase-mediated cross-feeding of AGOs might be associated with the *Bifidobacterium* community formation in their gut. The analysis showed that the relative abundance of the genus *Bifidobacterium* is significantly higher in the Endo/Exo-β1,3-Gal-ase-positive group than in the negative group (Fig. 5B, left panel). This trend was statistically significant even when the abundance of *B. vespertilionis*, the Endo/Exo-β1,3-Gal-ase-positive species in the microbiota, was subtracted from the total abundance of the genus *Bifidobacterium* (Fig. 5B, right panel). Endo/Exo-β1,3-Gal-ase-positive bifidobacteria may therefore contribute to syntrophic interactions within *Bifidobacterium* communities in the gut of gummivorous primates.

**Figure 5:**
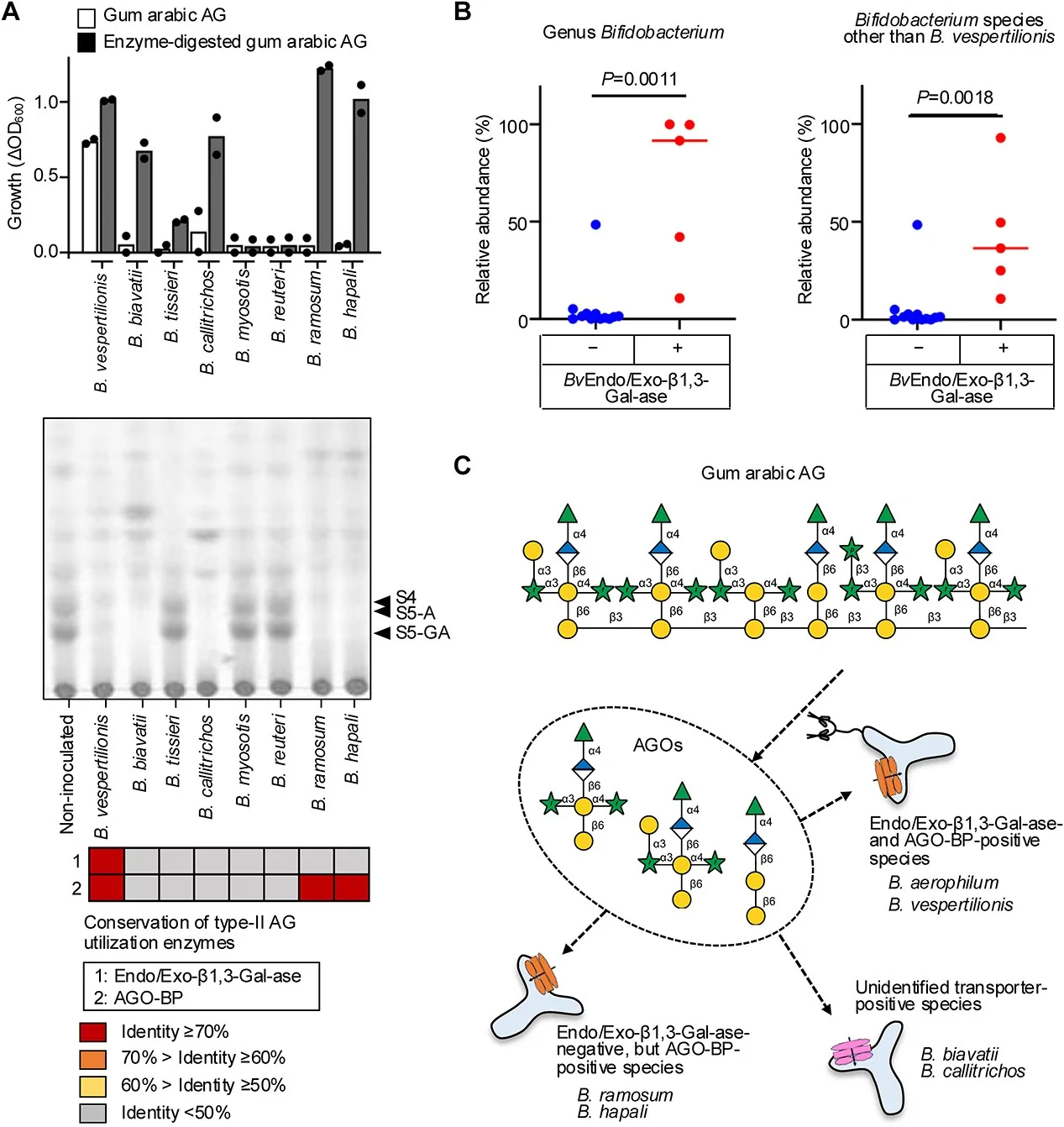
Potential syntrophic interactions mediated by Endo/Exo-β1,3-Gal-ase within *Bifidobacterium* communities in the gut of gummivorous primates. (A) (Upper panel) Growth of selected *Bifidobacterium* strains in medium supplemented with 1% gum arabic AG or *Ba*Endo/Exo-β1,3-Gal-ase-digested gum arabic AG (enzyme-digested gum arabic AG). AGO-BP-positive but Endo/Exo-β1,3-Gal-ase-negative species, double-negative species, and *B. vespertilionis* were selected for the analysis. All species except *B. ramosum* were detected in the metagenomic dataset (Fig. 4A). OD_600_ values obtained after 48 h of cultivation were corrected by subtracting the values obtained in the same medium without sugar supplementation (ΔOD_600_). Data obtained from biological duplicates were plotted, with bars representing the mean. (Lower panel) TLC analysis of sugars in the spent media supplemented with enzyme-digested gum arabic AG. The same samples used for OD_600_ measurements were spotted, along with a non-inoculated control. The presence of proteins orthologous to *Ba*Endo/Exo-β1,3-Gal-ase (lane 1) and *Ba*AGO-BP (lane 2) is indicated, with amino acid sequence identity levels relative to those of *B. aerophilum* shown by different colours. (B) Comparison of the relative abundances (%) of the genus *Bifidobacterium* (left panel) and the *Bifidobacterium* species other than *B. vespertilionis* (right panel) between Endo/Exo-β1,3-Gal-ase-negative (−, *n* = 12) and positive (+, *n* = 5) groups, in the metagenomic datasets. The number of hits per million reads in each dataset (for the presence of Endo/Exo-β1,3-Gal-ase), together with the relative abundance of each *Bifidobacterium* species, is used for the analysis (Fig. 4A and Supplementary Table S6). Reads corresponding to *B. vespertilionis* Endo/Exo-β1,3-Gal-ase were identified by mapping processed metagenomic reads (≥31 bp) to the DNA sequence of the enzyme, using thresholds of both >90% sequence identity and >90% coverage. Statistical significance was assessed using the two-sided Mann–Whitney *U* test. (C) A proposed model for Endo/Exo-β1,3-Gal-ase-mediated syntrophic interactions within *Bifidobacterium* communities in the gut of gummivorous primates. Gum arabic AG was used to depict the model, although its side chain structures may differ from those of natural gums consumed by native callitrichid (New World) monkeys. Scissors indicate Endo/Exo-β1,3-Gal-ase. Identified and unidentified AGO transporters are shown in different colours.

## Discussion

Dietary shifts can alter the composition of gut microbiota within a day by imposing selective pressure on gut microbes through changes in the availability of undigested dietary residues, primarily carbohydrates [94, 95]. Such compositional changes arise from relative fitness differences among microbial members within the community; however, the changes are often episodic rather than continuous, because host diets are usually not constant over time. Even when mutations or gene acquisitions arise in certain microbial species and confer a growth advantage under certain dietary conditions, such traits are unlikely to be retained as advantageous traits of the species because of the fluctuating nature of the diet. However, if a particular dietary pattern is maintained across generations in a host species, such mutations or gene acquisitions could persist in the host gut microbiome.

In this study, we identified highly efficient type-II AG assimilation pathways in *Bifidobacterium* species inhabiting the gut of gummivorous primates. The pathways comprises two key components, Endo/Exo-β1,3-Gal-ase and/or AGO transporter, namely, these two components do not always co-occur. Among the examined 99 *Bifidobacterium* species isolated from various sources, only two species—*B. aerophilum* and *B. vespertilionis*—are double positive and six species harbour the AGO transporter only (Supplementary Tables S5 and S12). No species possessing Endo/Exo-β1,3-Gal-ase alone was found in our analysis. The limited distribution of Endo/Exo-β1,3-Gal-ase suggested that these species contribute to cross-feeding interactions within *Bifidobacterium* communities, thereby increasing the relative abundance of bifidobacteria *in vivo*. In the faecal metagenomic datasets derived from golden lion tamarins and marmosets, the relative abundance of the genus *Bifidobacterium* was higher in the Endo/Exo-β1,3-Gal-ase-positive group than in the negative group (Fig. 5B, Supplementary Table S12, Fig. S6). This trend was observed even after excluding *B. vespertilionis*, the double-positive species detected in the datasets, from the calculation of the total microbiota abundance. Although these results should be interpreted with caution because the metagenomic reads were derived from pooled samples from multiple individuals, the data indicate that the presence of bifidobacteria capable of degrading the β-1,3-galactan backbone can promote the growth of other bifidobacterial species through cross-feeding interactions in the gut of gummivorous primates. The mechanism underlying cross-feeding was not fully elucidated in this study, because the cultivation experiments using gum arabic AG predigested with Endo/Exo-β1,3-Gal-ase revealed the presence of yet-unidentified AGO transporter(s) in several *Bifidobacterium* species (Fig. 5A). Moreover, the unidentified AGO transporter(s) might be more relevant to the cross-feeding interactions in the community, because *B. hapali*, a species harbouring the identified AGO transporter, was detected in only four of 17 metagenomic datasets and its relative abundance was less than 1.2% of the total microbiota in the four datasets (Fig. 4 and Supplementary Table S6). We were unable to examine whether all *Bifidobacterium* species detected in the datasets import AGOs released by Endo/Exo-β1,3-Gal-ase, because several species were not available from culture collections. Regardless of the identity of AGO transporters, the type-II AG assimilation pathways operating in these *Bifidobacterium* species appears to be more efficient than the other AG assimilation pathways identified so far (Supplementary Fig. S1), because AGO transporters, including the unidentified one, appear to be capable of internalizing at least up to a heptasaccharide (oligosaccharide S5-GA). Importing longer oligosaccharides can provide a competitive advantage for bacterial species to dominate the community, not only by reducing the energy expenditure required for transportation but also by enabling them to scavenge available resources in the environment, as exemplified by *B. infantis* which exploits an array of ABC transporters for the uptake of intact HMOs, thereby dominating the infant gut microbial community [15]. Apart from AGO transporters, the bifunctionality of Endo/Exo-β1,3-Gal-ase may also contribute to the efficient degradation of type-II AG by bifidobacteria. A cellulase from *Caldicellulosiruptor bescii*, CelA, which harbours both endo- and exo-acting domains within a single polypeptide, exhibits enhanced activity due to a proximity effect [96]. Efficient hydrolysis of type-II AG by Endo/Exo-β1,3-Gal-ase also stems from its relaxed specificity towards side chain modifications and cooperative effects between the two domains as mentioned. Whereas Exo-β1,3-Gal-ase from *B. longum* is unable to hydrolyse the β-1,3-galactan backbone without prior removal of α-d-Gal-(1 → 3)-l-Ara, β-l-Ara*p*-(1 → 3)-l-Ara, and Ara moieties [21], both of the Domains A and B of *Ba*Endo/Exo-β1,3-Gal-ase act on intact gum arabic AG. Neither *B. aerophilum* nor *B. vespertilionis* possesses extracellular enzymes required to release mono- and disaccharides from the intact AGs. Considering the limited occurrence of bifunctional Endo/Exo-β1,3-Gal-ase and AGO transporters in certain *Bifidobacterium* species, the type-II AG assimilation pathways identified in this study may have evolved or have been acquired after the diversification of primates into callitrichids, although an ancestral GH43_24 could have been shared among several *Bifidobacterium* species before the diversification of their hosts. Phylogenetic analysis also revealed that a predicted β-1,3-galactanase with a two-domain architecture is encoded in the genome of a plant-associated bacterium. Although it remains unclear whether this architecture evolved independently in the two organisms or was acquired through horizontal gene transfer between them, both bacteria appear to have inhabited environments rich in type-II AG.

A limitation of this study is the use of gum arabic AG as a model substrate for enzyme assays and bacterial cultivation. Gum arabic is derived from *A. senegal*, a tree native to regions in sub-Saharan Africa, and therefore does not represent the natural diet of the New World monkeys that, according to the observational studies, feed on a broader range of tree species belonging to the families Fabaceae (legume family) and Anacardiaceae (cashew family) found in their habitats [18, 97]. Given that the side chain structures of type-II AG vary considerably depending on plant source, future studies should require the use of AGs obtained from trees native to the New World. Despite this limitation, our findings may provide insights for the care of captive gummivorous primates, particularly marmosets, in which inflammatory bowel disease associated with an altered gut microbiota is prevalent [19, 26] and for which dietary supplementation with gum arabic has been recommended [25, 98]. Four out of six AGO-assimilating *Bifidobacterium* species—*B. aerophilum, B. callitrichos, B. hapali*, and *B. ramosum*—possess a protein orthologous (>88.3% identity) to *B. infantis* aromatic lactate dehydrogenase (Aldh) that catalyses the formation of indole-3-lactic acid (ILA) from indole-3-pyruvic acid, a deaminated form of tryptophan (Trp) [8, 51]. ILA is considered to play important roles in humans, particularly in the infant gut, by acting as an agonist of aryl hydrocarbon receptor (AhR) which is central to maintaining a barrier function of intestinal epithelia and suppressing excessive inflammatory responses [99]. An orthologous protein with >93.1% identity to human AhR is conserved in the common marmoset (*C. jacchus*). The genomes of the four Aldh-positive *Bifidobacterium* strains also encode the gene for tryptophan synthase β subunit (TrpB) (>94.0% identity to *B. infantis* TrpB). Aldh and TrpB are the core enzymes of the indole–ILA conversion pathway [100], in which indole released from Trp by the action of tryptophan indole-lyase (TIL) is first salvaged by TrpB to synthesize Trp by condensation with serine and subsequently converted to ILA by Aldh after deamination. Although this indole salvage pathway has been demonstrated only by *in vitro* experiments, given that indole serves as a precursor of the uremic toxin (indoxyl sulfate) and that TIL is widely present among gut microbes, the presence of the indole–ILA conversion pathway in *Bifidobacterium* species inhabiting the gummivorous primate gut may represent a symbiotic relationship acquired during the evolution of primates. Our results highlight the importance of further studies to identify and elucidate unique molecular strategies employed by symbionts, to better understand the evolutionary trajectories between hosts and the gut microbes, mediated through diversified host dietary habits.

## Supplementary Material

supplementary_files_wrag210

## Data Availability

The BioProject, BioSample, and DRA/SRA accession numbers for genomic sequencing of *B. vespertilionis* NBRC 113380^T^ are PRJDB37795, SAMD01695301, and DRR787059–DRR787060, respectively. The GenBank accession numbers for the complete genome sequence are AP044776 and AP044777.
