# Supplementary material for: Adaptations of gummivorous primate gut-associated bifidobacteria to type-II arabinogalactan utilization
Source: ISME J. 2026 Aug 19;20(1):wrag210. doi: 10.1093/ismejo/wrag210 (PMC13712309; doi:10.1093/ismejo/wrag210)
Supplement: Supplementary file 1 [file supplementary_files_wrag210.zip › Supplementary information_Fig. S1.pdf]

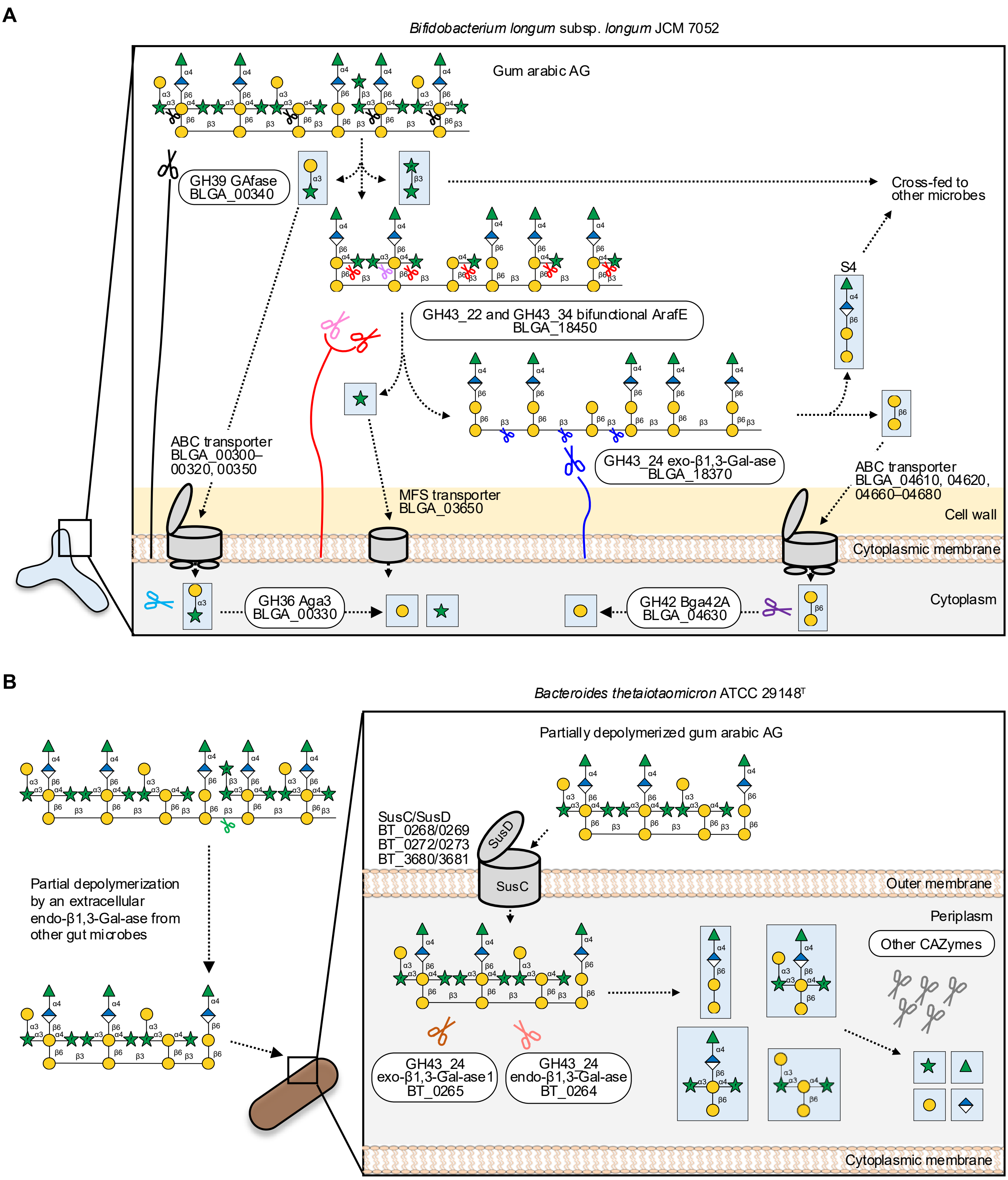

Supplementary Figure S1. Schematic representation of two distinct pathways for gum arabic AG utilization by *Bifidobacterium longum* subsp. *longum* JCM 7052 and *Bacteroides thetaiotaomicron* ATCC 29148^T.

(**A**) In the gum arabic AG assimilation pathway of *B. longum* JCM 7052, the side-chain disaccharide units, α-Gal-(1→3)-α-L-Ara*f*-(1→3)-*O*-R and β-L-Ara*p*-(1→3)-α-L-Ara*f*-(1→3)-*O*-R, are first hydrolysed by the GH39 3-*O*-α-D-galactosyl-α-L-arabinofuranosidase (GAfase) [6]. The bifunctional enzyme ArafE, which harbors GH43_22 and GH43_34 domains specific for α-1,3-Ara*f* and α-1,4-Ara*f* linkages, respectively, then releases L-Ara from the GAfase-trimmed AG [9]. Subsequently, the GH43_24 exo-β1,3-Gal-ase partially degrades the β-1,3-galactan backbone (main chain), generating galactobiose, β-Gal-(1→6)-Gal, and oligosaccharide S4. The released L-Ara, α-Gal-(1→3)-α-L-Ara, and β-Gal-(1→6)-Gal are imported by dedicated transporters and metabolized [3], whereas β-L-Ara*p*-(1→3)-α-L-Ara and S4 are not utilized and are instead cross-fed to other microbes. (**B**) *Ba. thetaiotaomicron* lacks a cell-surface endo-β1,3-Gal-ase and therefore can utilize only partially depolymerized gum arabic AG, which is generated by other symbionts such as *Bacteroides cellulosilyticus*. The depolymerized AG is imported via the SusCD system, and its β-1,3-galactan backbone is hydrolysed in the periplasm by the concerted action of Endo-β1,3-Gal-ase and Exo-β1,3-Gal-ase 1, followed by further degradation by CAZymes (GHs and a PL) into monosaccharides [4]. Transporters have not been biochemically characterized, and therefore their functions were predicted based on the genomic loci and transcriptome analyses [12, 13].
