# Supplementary material for: Adaptations of gummivorous primate gut-associated bifidobacteria to type-II arabinogalactan utilization
Source: ISME J. 2026 Aug 19;20(1):wrag210. doi: 10.1093/ismejo/wrag210 (PMC13712309; doi:10.1093/ismejo/wrag210)
Supplement: Supplementary file 1 [file supplementary_files_wrag210.zip › Supplementary information_Fig. S2.pdf]

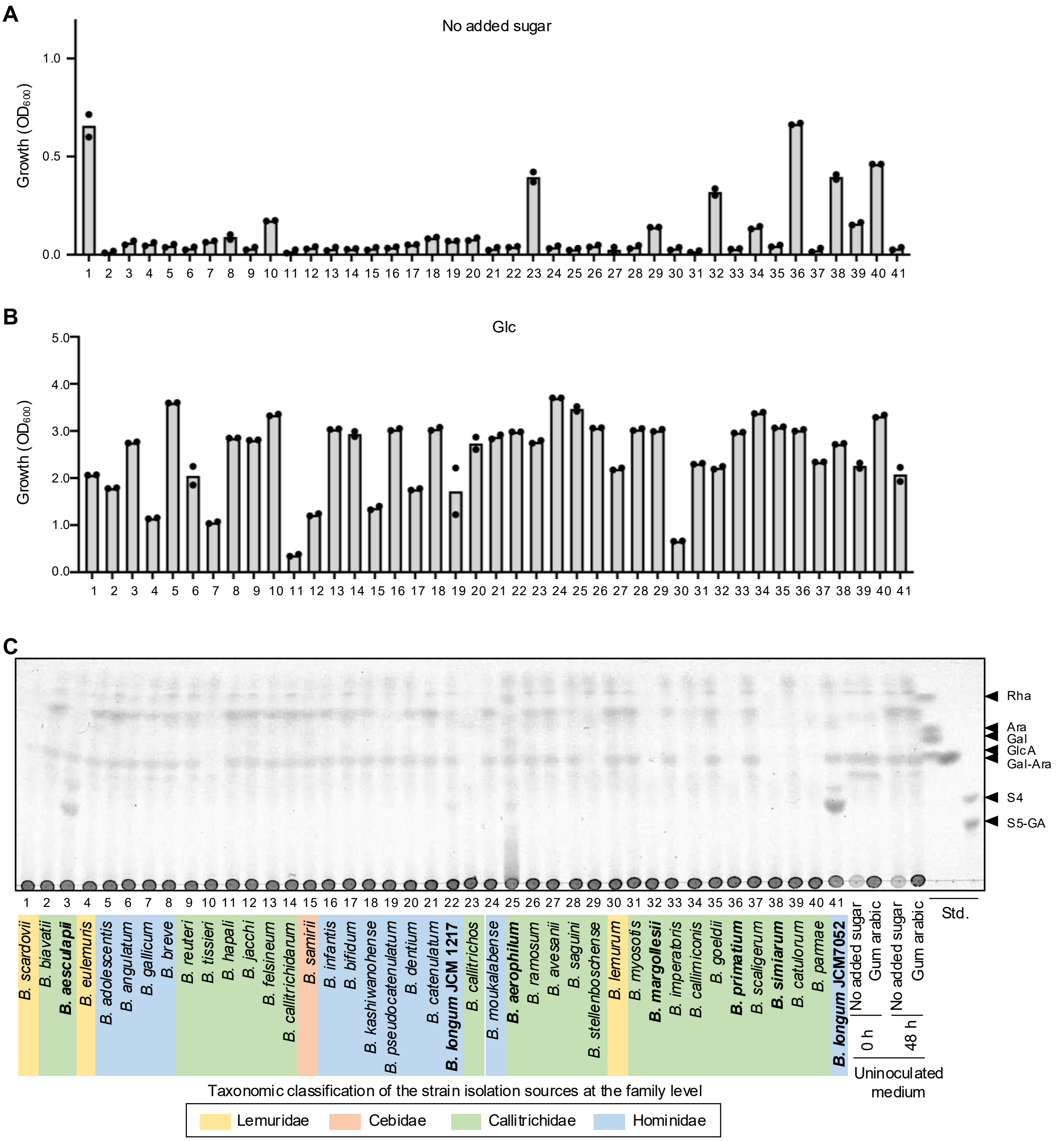

Supplementary Figure S2. Growth and sugar utilization of various primate gut-associated *Bifidobacterium* species. (**A** and **B**) OD_{600} values after 48 h of cultivation in medium without (**A**) or with glucose (Glc) (**B**). Values obtained from biological duplicates were plotted, with bars representing the mean. (**C**) TLC analysis of sugars in culture supernatants collected after 48 h of cultivation in medium supplemented with 1% gum arabic AG (**Fig. 1A**). A representative result from biological duplicates is shown. Non-inoculated medium was used as a control. Standard sugars used are L-rhamnose (Rha), L-arabinose (Ara), galactose (Gal), glucuronic acid (GlcA), α-Gal-(1→3)-L-Ara (Gal-Ara), S4, and S5-GA. The species names and their isolation sources (family-level classification of host primates) are indicated (see also **Supplementary Table S1**). *Bifidobacterium* species referred to in the text are shown in bold. Sample order differs from that shown in **Fig. 1A**.
