# Supplementary material for: Adaptations of gummivorous primate gut-associated bifidobacteria to type-II arabinogalactan utilization
Source: ISME J. 2026 Aug 19;20(1):wrag210. doi: 10.1093/ismejo/wrag210 (PMC13712309; doi:10.1093/ismejo/wrag210)
Supplement: Supplementary file 1 [file supplementary_files_wrag210.zip › Supplementary information_Fig. S3.pdf]

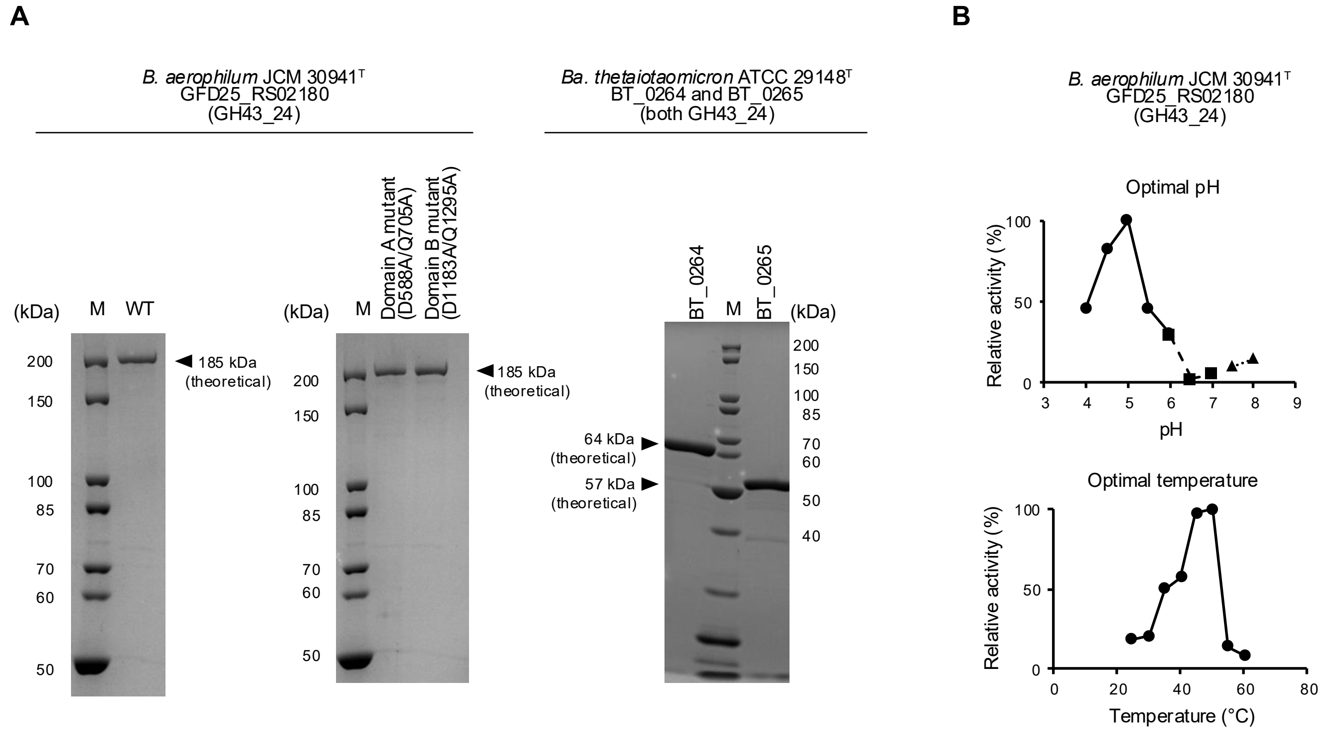

Supplementary Figure S3. **Purification and characterization of GFD25_RS02180 (GH43_24) from *B. aerophilum* JCM 30941^{T}**.

(**A**) SDS-PAGE of purified recombinant wild-type (WT) protein and Domain A- and Domain B mutants of GFD25_RS02180 (GH43_24). Endo-β1,3-Gal-ase (BT_0264) and exo-β1,3-Gal-ase 1 (BT_0265) from *Ba. thetaiotaomicron* ATCC 29148 were also prepared for comparison of gum arabic AG hydrolysis activity. Proteins were stained with Coomassie Brilliant Blue R-250. M indicates the molecular size marker. (**B**) Optimal pH and temperature of the GFD25_RS02180 protein for the hydrolysis of gum arabic AG. Sodium acetate buffer (closed circles), MES buffer (closed squares), and HEPES buffer (closed triangles) were used to determine the optimal pH. Activity is expressed as the percent of that obtained at pH 5.0. Optimal temperature was assessed at pH 5.0. The activity relative to that at 50ºC is shown.
