# Supplementary material for: Adaptations of gummivorous primate gut-associated bifidobacteria to type-II arabinogalactan utilization
Source: ISME J. 2026 Aug 19;20(1):wrag210. doi: 10.1093/ismejo/wrag210 (PMC13712309; doi:10.1093/ismejo/wrag210)
Supplement: Supplementary file 1 [file supplementary_files_wrag210.zip › Supplementary information_Fig. S4.pdf]

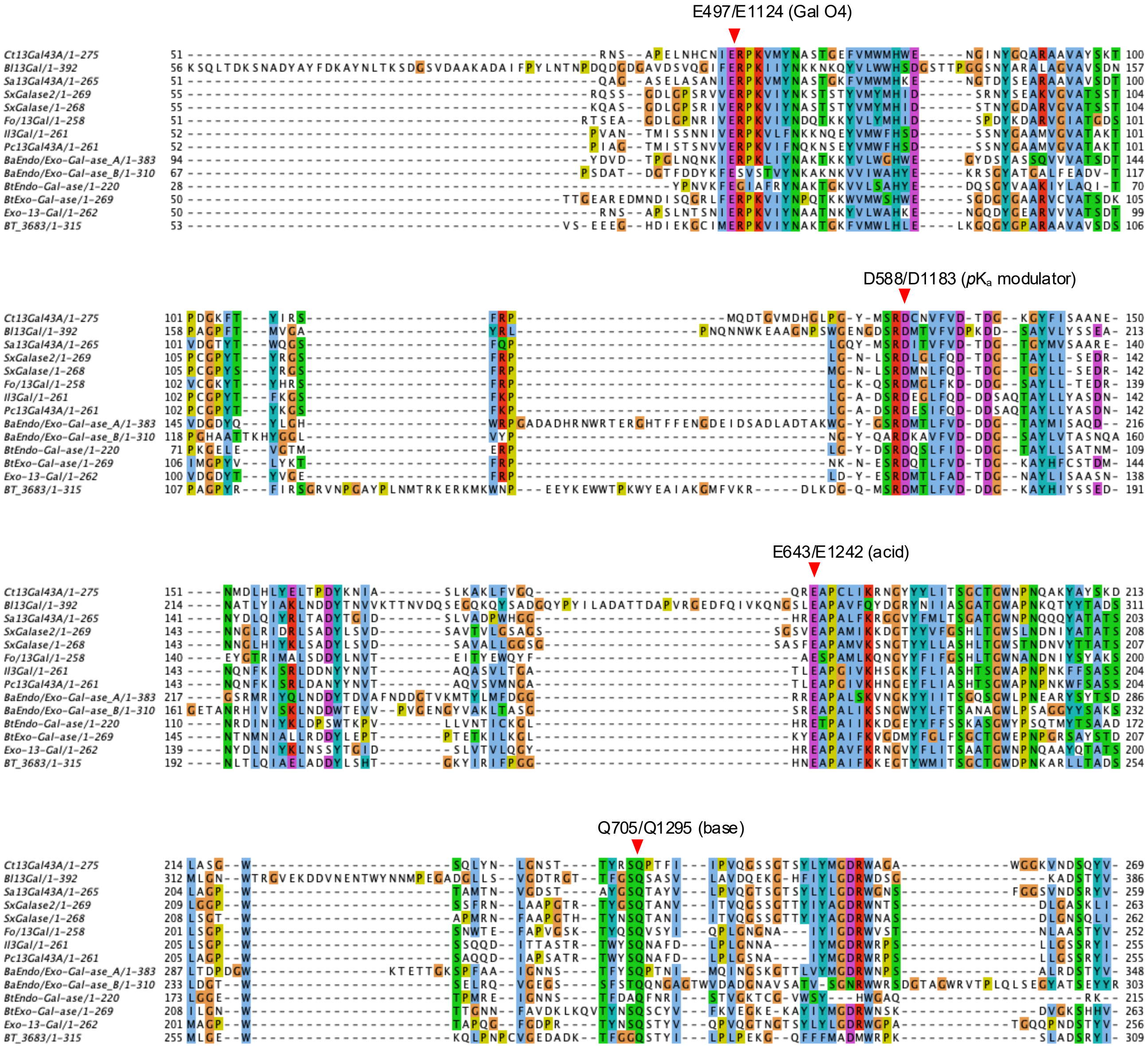

Supplementary Figure S4. **Multiple amino acid sequence alignment of characterized members of GH43_24 with Domain A and Domain B of *B. aerophilum* GFD25_RS02180.**

The amino acid sequences of the two GH43_24 domains (Domain A and Domain B) from *B. aerophilum* GFD25_RS02180 were aligned with those of previously characterized GH43_24 endo- and exo-β-1,3-galactanases [4, 5, 14–20]. The sequences of the GH43_24 domains were extracted using InterPro [21]. Alignment was generated using Jalview 2.11.5.0 [22]. Catalytically important residues identified in *Bt*Exo-β1,3-Gal-ase 2 (BT_3683) [4] are marked by red triangles, and their roles in the catalysis are indicated. The corresponding residue numbers of GFD25_RS02180 are indicated. Accession numbers and pairwise sequence identities of these enzymes are provided in Supplementary Information (**Supplementary Table S3**).
