# Supplementary material for: Adaptations of gummivorous primate gut-associated bifidobacteria to type-II arabinogalactan utilization
Source: ISME J. 2026 Aug 19;20(1):wrag210. doi: 10.1093/ismejo/wrag210 (PMC13712309; doi:10.1093/ismejo/wrag210)
Supplement: Supplementary file 1 [file supplementary_files_wrag210.zip › Supplementary information_Fig. S5.pdf]

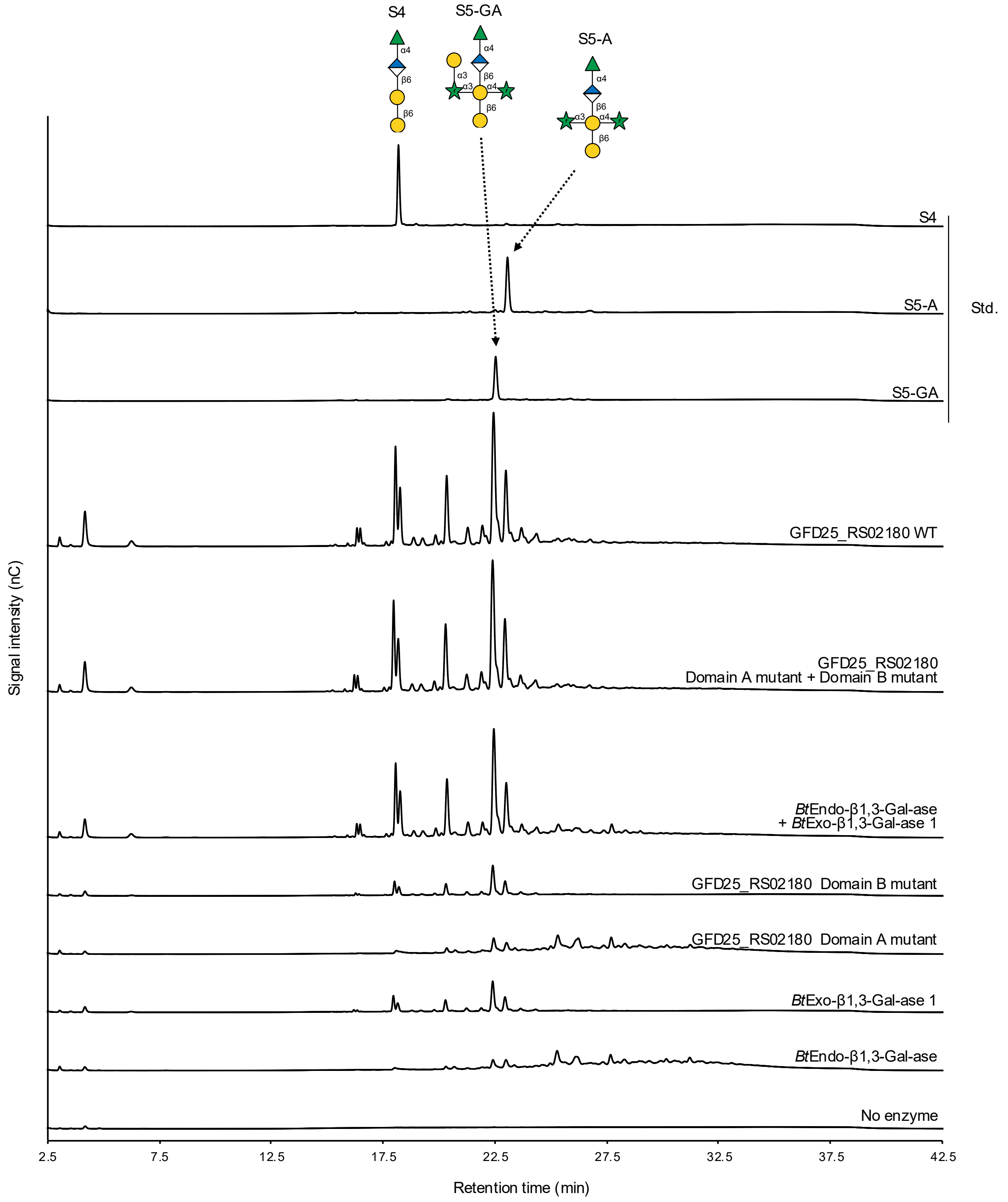

Supplementary Figure S5. Dual exo- and endo modes of action of β-1,3-galactanase of *B. aerophilum* GFD25_RS02180 (Endo/Exo-β1,3-Gal-ase).
Reaction products obtained after incubating gum arabic AG (1%) with purified wild-type (WT) enzyme, Domain A mutant (D588A/Q705A), Domain B mutant (D1183A/Q1295A), or a combination of the two mutants (each at 20 nM) for 24 h(**Fig. 2D**) were analysed using HPAEC-PAD. *Bt*Endo-β1,3-Gal-ase and *Bt*Exo-β1,3-Gal-ase 1 were also included in the analysis for comparison. For standard sugars (S4, S5-A, and S5-GA), the same chromatograms as in **Fig. 2C** were used.
