# Supplementary material for: Adaptations of gummivorous primate gut-associated bifidobacteria to type-II arabinogalactan utilization
Source: ISME J. 2026 Aug 19;20(1):wrag210. doi: 10.1093/ismejo/wrag210 (PMC13712309; doi:10.1093/ismejo/wrag210)
Supplement: Supplementary file 1 [file supplementary_files_wrag210.zip › Supplementary information_Fig. S6.pdf]

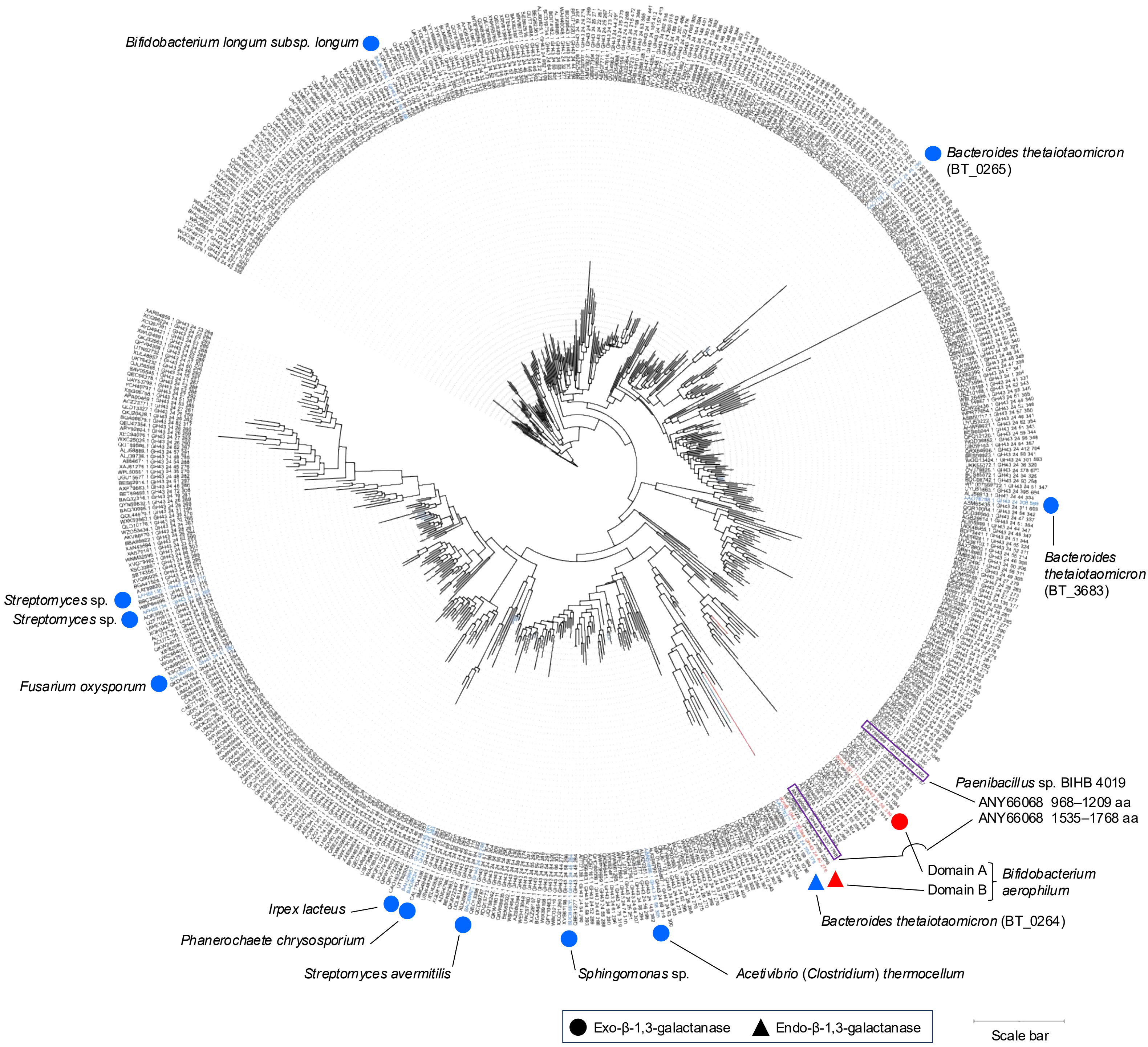

Supplementary Figure S6. **Phylogenetic tree of representative GH43_24 members.**
Phylogenetic analysis was performed using 508 non-redundant representative GH43_24 protein sequences retrieved from the dbCAN entries following CD-HIT clustering [23] at a 70% sequence identity threshold. Sequences shorter than 200 amino acids were excluded from the analysis, except for one characterized enzyme. Accession numbers are provided in Supplementary Information (**Supplementary Table S4**). Multiple sequence alignment was performed using MAFFT v7.505 [24], followed by maximum-likelihood tree construction using IQ-TREE v2.0.7 [25] and visualization using iTOL v7 [26]. Blue and red symbols indicate previously characterized enzymes [4, 5, 14–20] and Domain A and Domain B of *Ba*Endo/Exo-Gal-ase, respectively. The two GH43_24 domains in ANY66068 from *Paenibacillus* sp. BIHB 4019 are enclosed by purple boxes. The source organism of each enzyme mentioned in the text is indicated. Circles and triangles denote confirmed exo- and endo-mode β-1,3-galactanase activities, respectively. The scale bar indicates 1 substitution per site.
