# Supplementary material for: Adaptations of gummivorous primate gut-associated bifidobacteria to type-II arabinogalactan utilization
Source: ISME J. 2026 Aug 19;20(1):wrag210. doi: 10.1093/ismejo/wrag210 (PMC13712309; doi:10.1093/ismejo/wrag210)
Supplement: Supplementary file 1 [file supplementary_files_wrag210.zip › Supplementary information_Fig. S7.pdf]

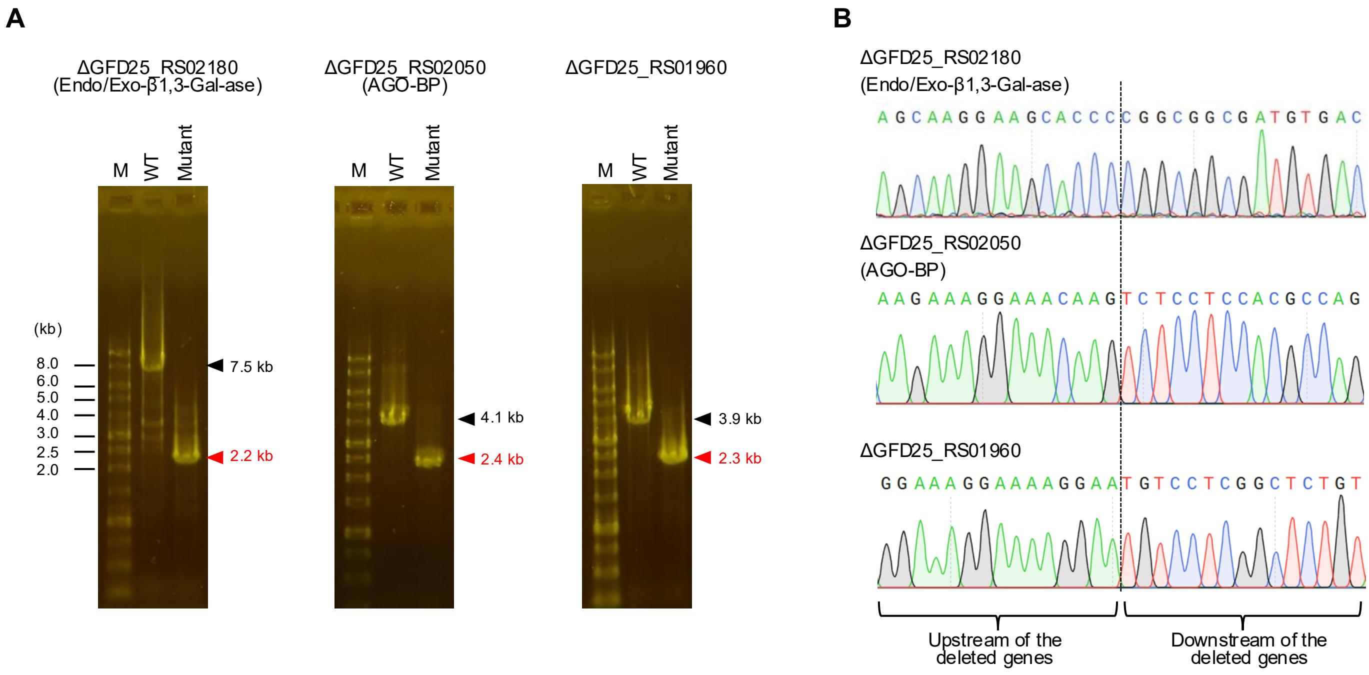

Supplementary Figure S7. Scarless gene deletions in the genome of *B. aerophilum*.
(**A**) Results of agarose gel electrophoresis of genomic PCR products. PCR was performed using primer pairs designed to anneal outside the regions used for homologous recombination (**Supplementary Table S2**). Estimated lengths of PCR products for the WT (black) and mutant (red) strains are indicated. (**B**) Sanger sequencing electropherograms obtained for the amplified fragments. The dashed line marks the scarless deletion junction at the target sites.
