# Supplementary material for: Adaptations of gummivorous primate gut-associated bifidobacteria to type-II arabinogalactan utilization
Source: ISME J. 2026 Aug 19;20(1):wrag210. doi: 10.1093/ismejo/wrag210 (PMC13712309; doi:10.1093/ismejo/wrag210)
Supplement: Supplementary file 1 [file supplementary_files_wrag210.zip › Supplementary information_Fig. S8.pdf]

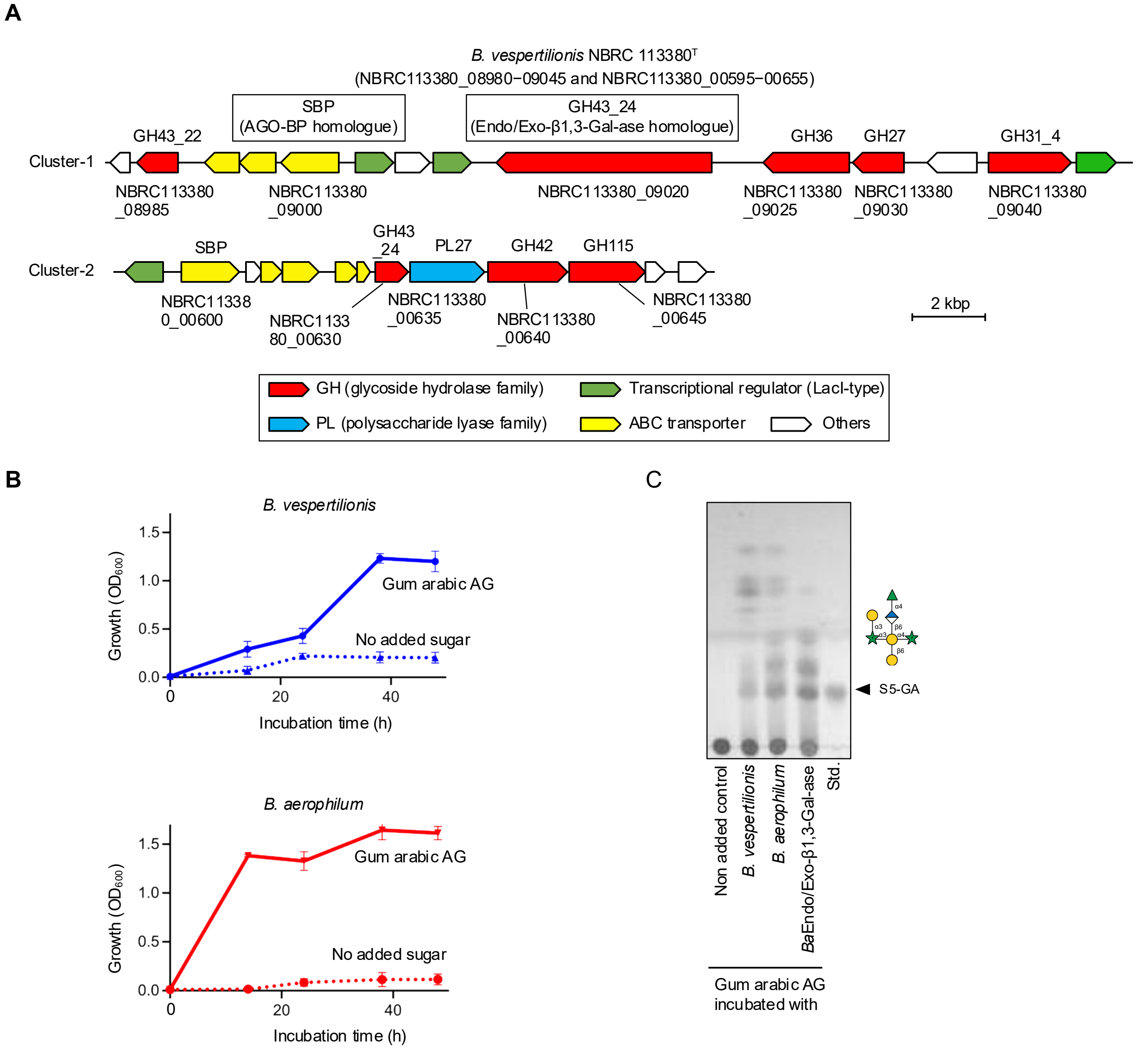

Supplementary Figure S8. Two gene clusters presumably involved in the type-II AG assimilation by *B. vespertilionis*, another species capable of assimilating gum arabic AG.

(**A**) Schematic representation of two gene clusters predicted to be involved in gum arabic AG utilization by *B. vespertilionis* NBRC 113380^T. These clusters were identified by blastp searches using the CAZymes and AGO-BP of *B. aerophilum* as queries. GHs and a PL were predicted by dbCAN3 [27] and are shown with their family (subfamily) numbers (**Supplementary Tables S10–S12**). The diagrams were created using drawGeneArrows3 (https://www.ige.tohoku.ac.jp/joho/labhome/tool.html). (**B**) Growth of *B. vespertilionis* NBRC 113380^T (top panel) and *B. aerophilum* JCM 30941^T (bottom panel) in medium supplemented with or without 1% gum arabic AG. The samples were taken at the indicated time points for measuring OD_{600} values. Line plots with whiskers represent the mean ± SD of three independent experiments. (**C**) TLC analysis of reaction products following incubation of gum arabic AG with cell suspensions of *B. vespertilionis* NBRC 113380^T or *B. aerophilum* JCM 30941^T, or with purified recombinant *Ba*Endo/Exo-β1,3-Gal-ase. S5-GA was used as a standard sugar.
