# Supplementary material for: Adaptations of gummivorous primate gut-associated bifidobacteria to type-II arabinogalactan utilization
Source: ISME J. 2026 Aug 19;20(1):wrag210. doi: 10.1093/ismejo/wrag210 (PMC13712309; doi:10.1093/ismejo/wrag210)
Supplement: Supplementary file 1 [file supplementary_files_wrag210.zip › Supplementary information_all.zip!/Supplementary information_all/Supplementary information_all.pdf]

Supplementary Information for

Adaptations of gummivorous primate gut-associated bifidobacteria to type-II arabinogalactan utilization

Yuki Sasaki[1], Tomoya Kozakai[1], Moe Inoue[1], Mikiyasu Sakanaka[1], Toshihiko Katoh[1], Hiroki Kaneko[2], Hiroo Imai[3], Toshitaka Odamaki[1,2], Kiyotaka Fujita[4], and Takane Katayama[1]

[1]Graduate School of Biostudies, Kyoto University

[2]Biotics Research Institute, R & D Division, Morinaga Milk Industry Co., Ltd.

[3]Institute for the Evolutionary Origins of Human Behavior, Kyoto University

[4]Faculty of Agriculture, Kagoshima University, Kagoshima, Japan

Supplementary Tables are provided by a separate file.

Table S1. *Bifidobacterium* species (subspecies) used in this study, ordered as in Figure 1A except for *B. vespertilionis*.

Table S2. Primers used in this study.

Table S3. Amino acid sequence identities of the Domain A and Domain B regions of *B. aerophilum* GFD25_RS02180 to previously characterized exo- and endo-β-1,3-galactanases in the same GH subfamily, ordered by Domain A sequence identity (%) (excluding GFD25_RS02180 itself).

Table S4. Non-redundant GH43_24 protein sequences used for phylogenetic tree construction, ordered by GenBank accession.

Table S5. *Bifidobacterium* species and strains used for the gene conservation analysis, alphabetically ordered first by isolation source category and then by species (subspecies).

Table S6. Overview of the mining of deposited metagenomic datasets [2].

Table S7. dbCAN-predicted CAZymes in *B. aerophilum* JCM 30941, sorted first by signal peptide presence and then by GenBank accession.

Table S8. Genes located in the predicted type-II AG utilization cluster of *B. aerophilum* JCM 30941, ordered by locus tag.

Table S9. Overview of the complete genome sequence of *B. vespertilionis* NBRC 113380.

Table S10. dbCAN-predicted CAZymes of *B. vespertilionis* NBRC 113380, sorted first by signal peptide presence and then by GenBank accession.

Table S11. *B. vespertilionis* NBRC 113380 proteins orthologous to those encoded in the type-II AG utilization gene cluster of *B. aerophilum* JCM 30941, ordered by locus tag of *B. aerophilum*.

Table S12. Occurrence of proteins homologous to the CAZymes and AGO-BP encoded in the *B. aerophilum* type-II AG utilization gene cluster in the genomes of various *Bifidobacterium* species, ordered by prevalence.

Table S13. dbCAN-predicted CAZymes in *B. biavatii* JCM 17299 and *B. callitrichos* JCM 17296, each sorted first by signal peptide presence and then by GenBank accession.

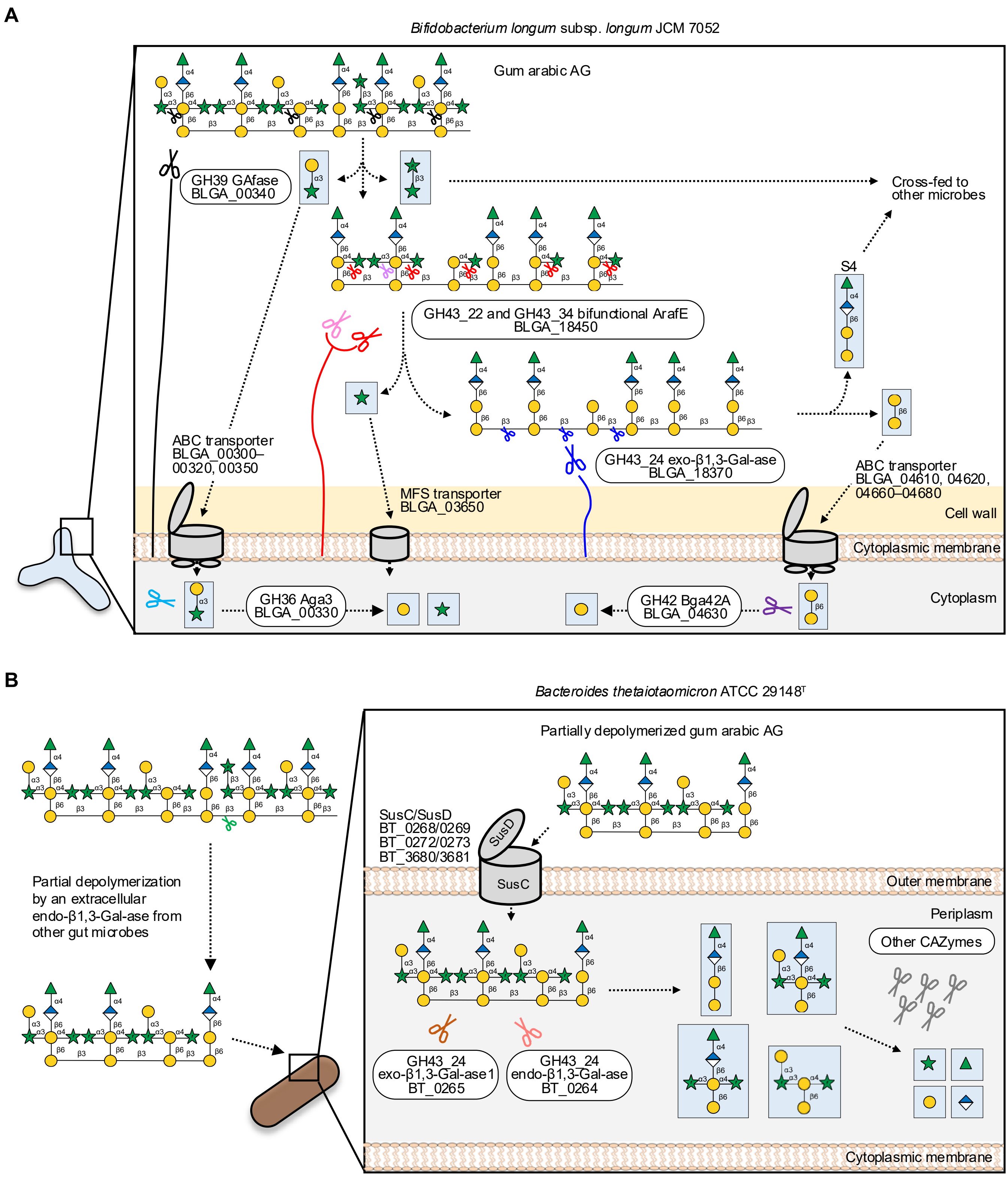

Supplementary Figure S1. Schematic representation of two distinct pathways for gum arabic AG utilization by *Bifidobacterium longum* subsp. *longum* JCM 7052 and *Bacteroides thetaiotaomicron* ATCC 29148^{T}.
(**A**) In the gum arabic AG assimilation pathway of *B. longum* JCM 7052, the side-chain disaccharide units, α-Gal-(1→3)-α-L-Ara*f*-(1→3)-*O*-R and β-L-Ara*p*-(1→3)-α-L-Ara*f*-(1→3)-*O*-R, are first hydrolysed by the GH39 3-*O*-α-D-galactosyl-α-L-arabinofuranosidase (GAfase) [6]. The bifunctional enzyme ArafE, which harbors GH43_22 and GH43_34 domains specific for α-1,3-Ara*f* and α-1,4-Ara*f* linkages, respectively, then releases L-Ara from the GAfase-trimmed AG [9]. Subsequently, the GH43_24 exo-β1,3-Gal-ase partially degrades the β-1,3-galactan backbone (main chain), generating galactobiose, β-Gal-(1→6)-Gal, and oligosaccharide S4. The released L-Ara, α-Gal-(1→3)-α-L-Ara, and β-Gal-(1→6)-Gal are imported by dedicated transporters and metabolized [3], whereas β-L-Ara*p*-(1→3)-α-L-Ara and S4 are not utilized and are instead cross-fed to other microbes. (**B**) *Ba. thetaiotaomicron* lacks a cell-surface endo-β1,3-Gal-ase and therefore can utilize only partially depolymerized gum arabic AG, which is generated by other symbionts such as *Bacteroides cellulosilyticus*. The depolymerized AG is imported via the SusCD system, and its β-1,3-galactan backbone is hydrolysed in the periplasm by the concerted action of Endo-β1,3-Gal-ase and Exo-β1,3-Gal-ase 1, followed by further degradation by CAZymes (GHs and a PL) into monosaccharides [4]. Transporters have not been biochemically characterized, and therefore their functions were predicted based on the genomic loci and transcriptome analyses [12, 13].

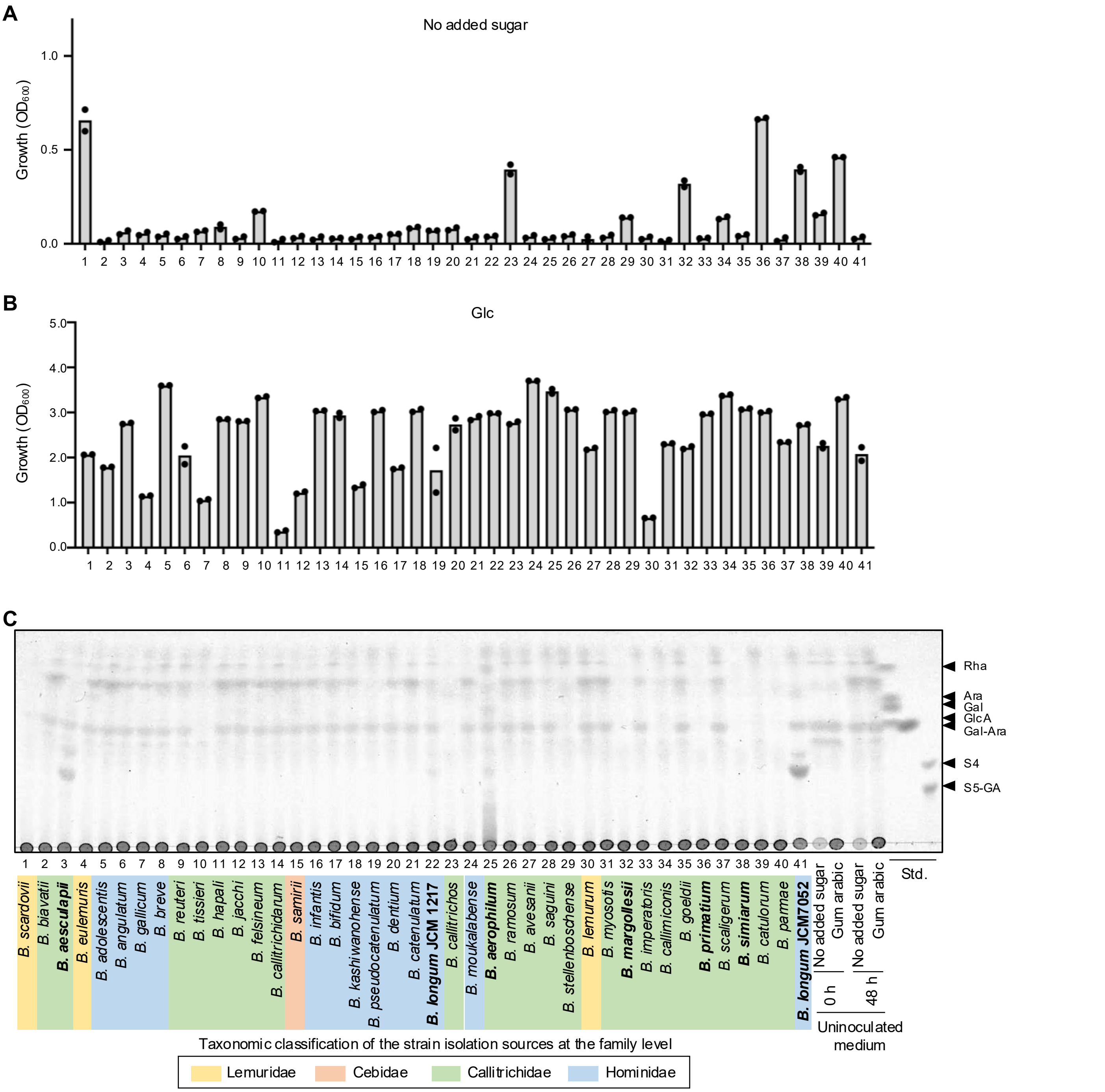

Supplementary Figure S2. Growth and sugar utilization of various primate gut-associated *Bifidobacterium* species. (**A** and **B**) OD_{600} values after 48 h of cultivation in medium without (**A**) or with glucose (Glc) (**B**). Values obtained from biological duplicates were plotted, with bars representing the mean. (**C**) TLC analysis of sugars in culture supernatants collected after 48 h of cultivation in medium supplemented with 1% gum arabic AG (**Fig. 1A**). A representative result from biological duplicates is shown. Non-inoculated medium was used as a control. Standard sugars used are L-rhamnose (Rha), L-arabinose (Ara), galactose (Gal), glucuronic acid (GlcA), α-Gal-(1→3)-L-Ara (Gal-Ara), S4, and S5-GA. The species names and their isolation sources (family-level classification of host primates) are indicated (see also **Supplementary Table S1**). *Bifidobacterium* species referred to in the text are shown in bold. Sample order differs from that shown in **Fig. 1A**.

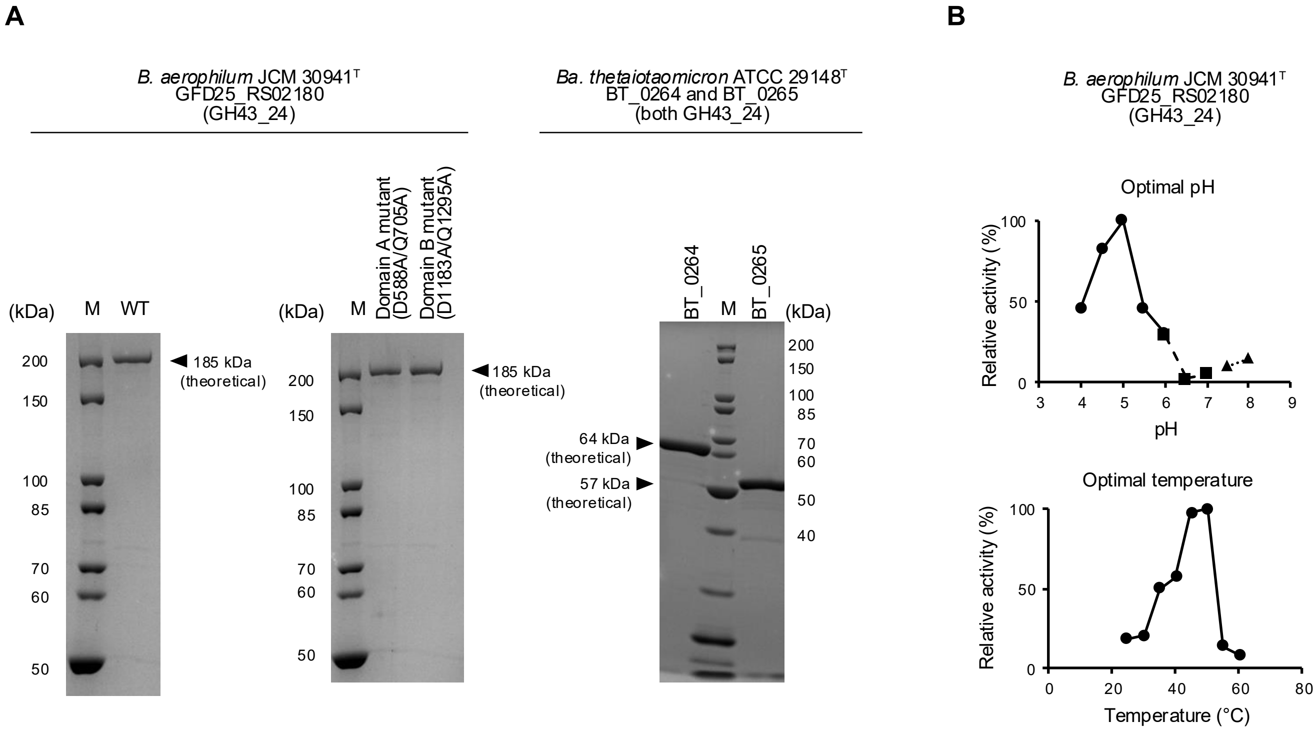

Supplementary Figure S3. **Purification and characterization of GFD25_RS02180 (GH43_24) from *B. aerophilum* JCM 30941^{T}**.

(**A**) SDS-PAGE of purified recombinant wild-type (WT) protein and Domain A- and Domain B mutants of GFD25_RS02180 (GH43_24). Endo-β1,3-Gal-ase (BT_0264) and exo-β1,3-Gal-ase 1 (BT_0265) from *Ba. thetaiotaomicron* ATCC 29148 were also prepared for comparison of gum arabic AG hydrolysis activity. Proteins were stained with Coomassie Brilliant Blue R-250. M indicates the molecular size marker. (**B**) Optimal pH and temperature of the GFD25_RS02180 protein for the hydrolysis of gum arabic AG. Sodium acetate buffer (closed circles), MES buffer (closed squares), and HEPES buffer (closed triangles) were used to determine the optimal pH. Activity is expressed as the percent of that obtained at pH 5.0. Optimal temperature was assessed at pH 5.0. The activity relative to that at 50ºC is shown.

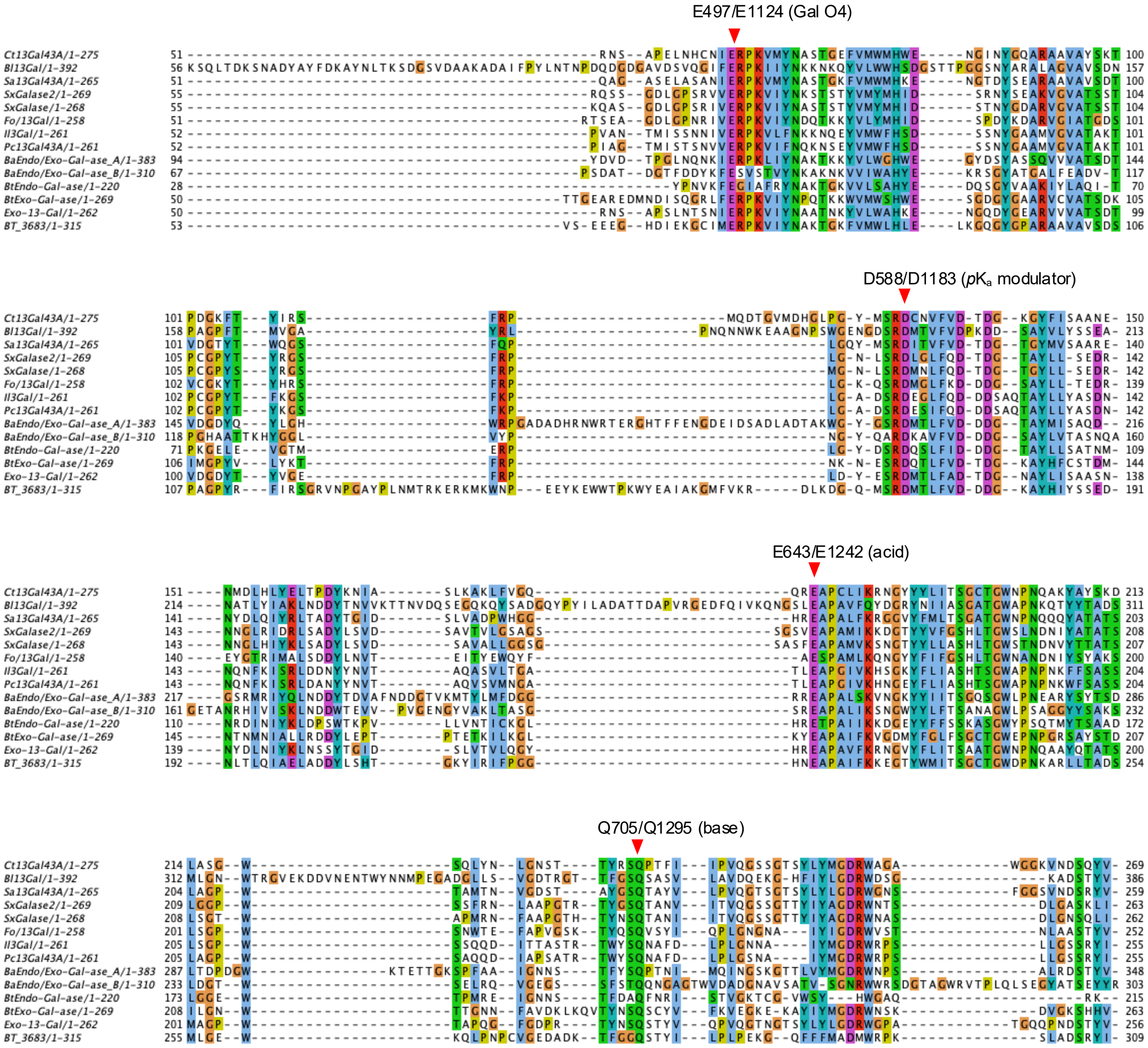

Supplementary Figure S4. **Multiple amino acid sequence alignment of characterized members of GH43_24 with Domain A and Domain B of *B. aerophilum* GFD25_RS02180.**

The amino acid sequences of the two GH43_24 domains (Domain A and Domain B) from *B. aerophilum* GFD25_RS02180 were aligned with those of previously characterized GH43_24 endo- and exo-β-1,3-galactanases [4, 5, 14–20]. The sequences of the GH43_24 domains were extracted using InterPro [21]. Alignment was generated using Jalview 2.11.5.0 [22]. Catalytically important residues identified in *Bt*Exo-β1,3-Gal-ase 2 (BT_3683) [4] are marked by red triangles, and their roles in the catalysis are indicated. The corresponding residue numbers of GFD25_RS02180 are indicated. Accession numbers and pairwise sequence identities of these enzymes are provided in Supplementary Information (**Supplementary Table S3**).

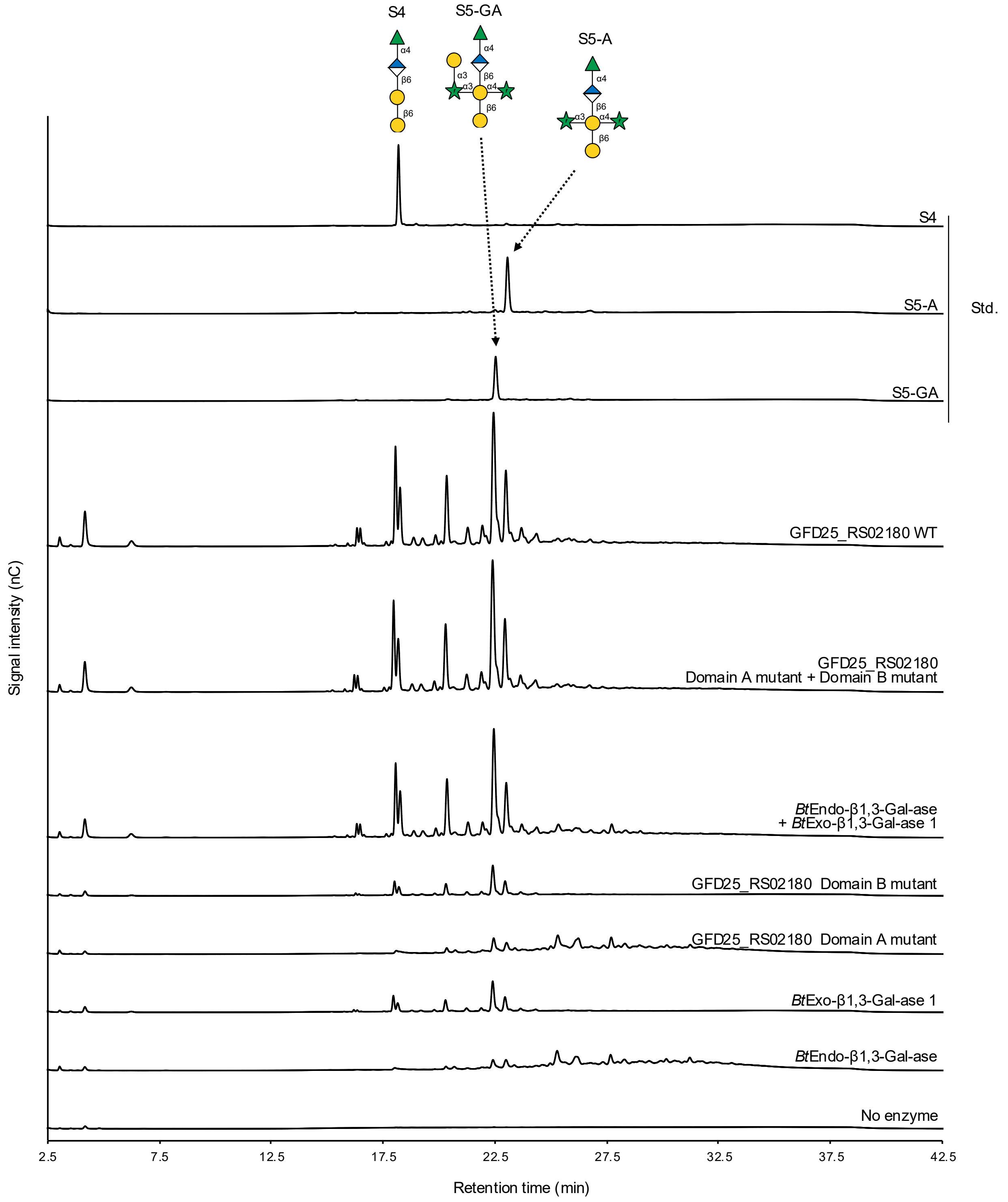

Supplementary Figure S5. Dual exo- and endo modes of action of β-1,3-galactanase of *B. aerophilum* GFD25_RS02180 (Endo/Exo-β1,3-Gal-ase).

Reaction products obtained after incubating gum arabic AG (1%) with purified wild-type (WT) enzyme, Domain A mutant (D588A/Q705A), Domain B mutant (D1183A/Q1295A), or a combination of the two mutants (each at 20 nM) for 24 h (**Fig. 2D**) were analysed using HPAEC-PAD. *Bt*Endo-β1,3-Gal-ase and *Bt*Exo-β1,3-Gal-ase 1 were also included in the analysis for comparison. For standard sugars (S4, S5-A, and S5-GA), the same chromatograms as in **Fig. 2C** were used.

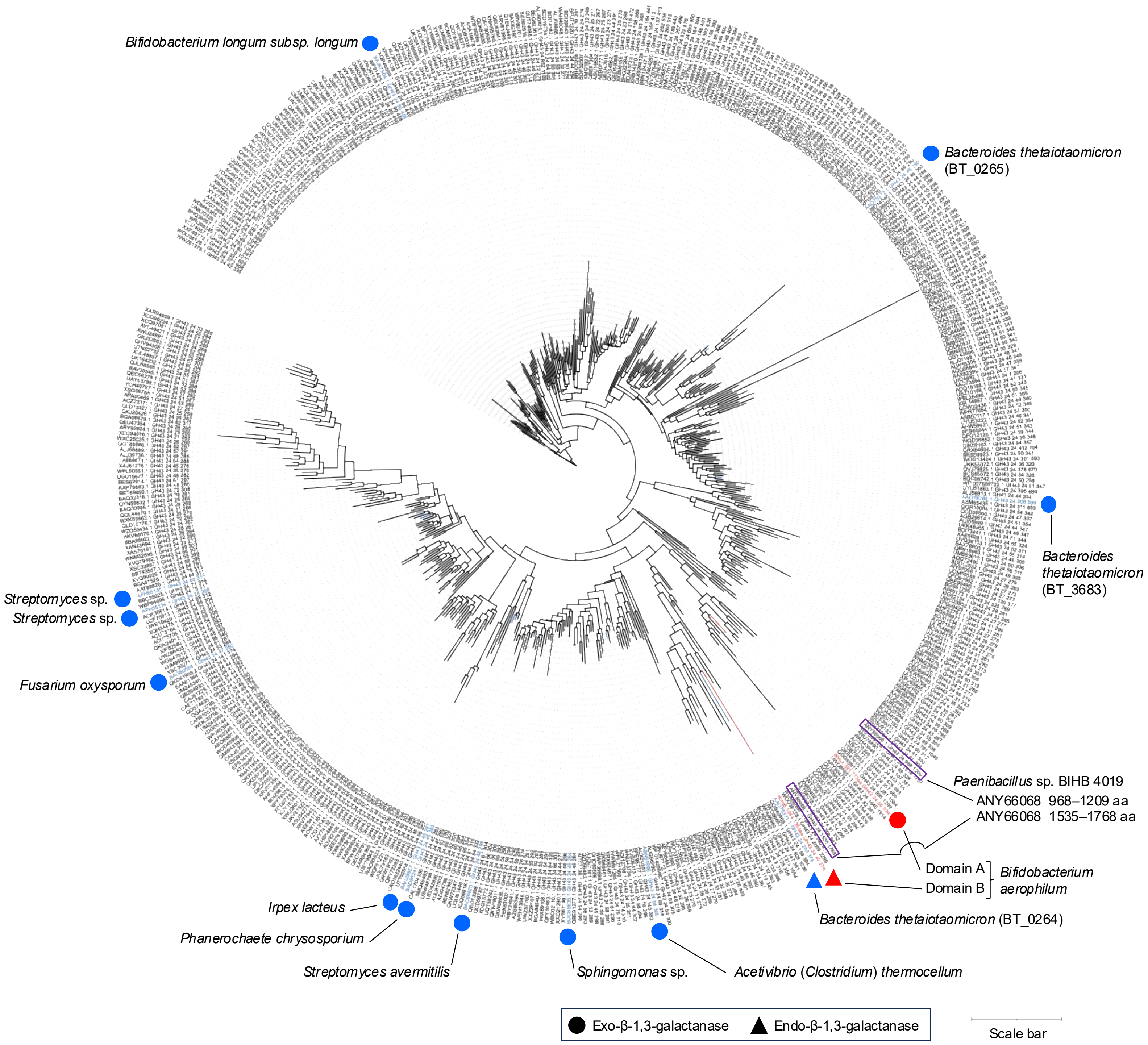

Supplementary Figure S6. **Phylogenetic tree of representative GH43_24 members.**
Phylogenetic analysis was performed using 508 non-redundant representative GH43_24 protein sequences retrieved from the dbCAN entries following CD-HIT clustering [23] at a 70% sequence identity threshold. Sequences shorter than 200 amino acids were excluded from the analysis, except for one characterized enzyme. Accession numbers are provided in Supplementary Information (**Supplementary Table S4**). Multiple sequence alignment was performed using MAFFT v7.505 [24], followed by maximum-likelihood tree construction using IQ-TREE v2.0.7 [25] and visualization using iTOL v7 [26]. Blue and red symbols indicate previously characterized enzymes [4, 5, 14–20] and Domain A and Domain B of *Ba*Endo/Exo-Gal-ase, respectively. The two GH43_24 domains in ANY66068 from *Paenibacillus* sp. BIHB 4019 are enclosed by purple boxes. The source organism of each enzyme mentioned in the text is indicated. Circles and triangles denote confirmed exo- and endo-mode β-1,3-galactanase activities, respectively. The scale bar indicates 1 substitution per site.

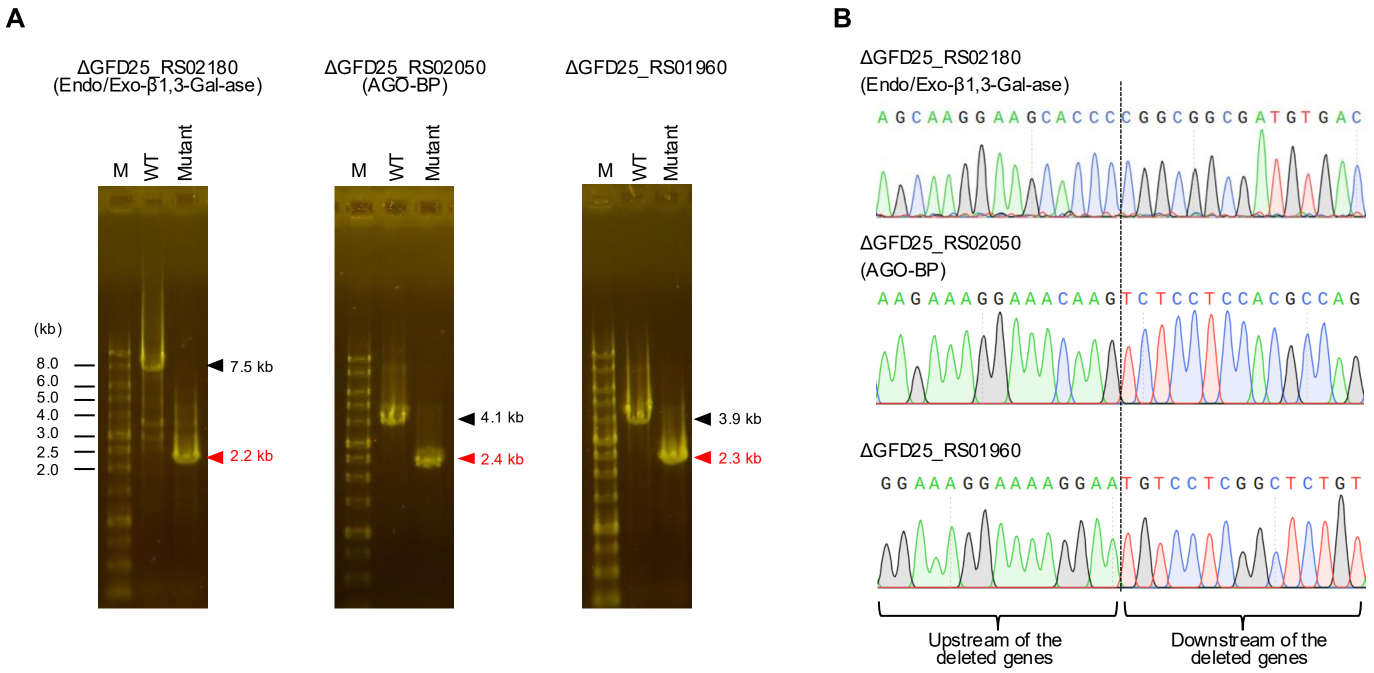

Supplementary Figure S7. Scarless gene deletions in the genome of *B. aerophilum*.
(**A**) Results of agarose gel electrophoresis of genomic PCR products. PCR was performed using primer pairs designed to anneal outside the regions used for homologous recombination (**Supplementary Table S2**). Estimated lengths of PCR products for the WT (black) and mutant (red) strains are indicated. (**B**) Sanger sequencing electropherograms obtained for the amplified fragments. The dashed line marks the scarless deletion junction at the target sites.

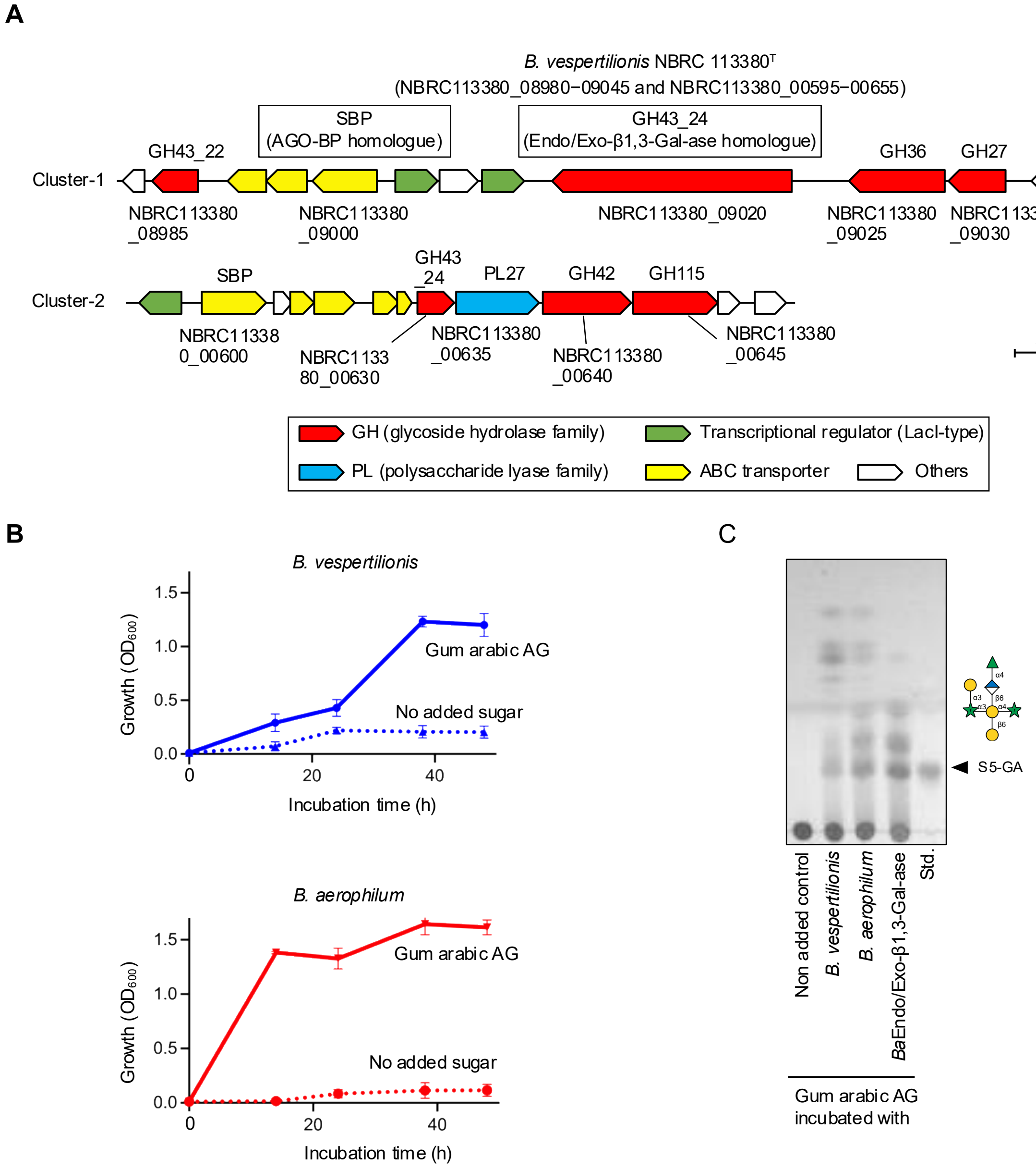

Supplementary Figure S8. Two gene clusters presumably involved in the type-II AG assimilation by *B. vespertilionis*, another species capable of assimilating gum arabic AG.
(**A**) Schematic representation of two gene clusters predicted to be involved in gum arabic AG utilization by *B. vespertilionis* NBRC 113380^{T}. These clusters were identified by blastp searches using the CAZymes and AGO-BP of *B. aerophilum* as queries. GHs and a PL were predicted by dbCAN3 [27] and are shown with their family (subfamily) numbers (**Supplementary Tables S10–S12**). The diagrams were created using drawGeneArrows3 (https://www.ige.tohoku.ac.jp/joho/labhome/tool.html). (**B**) Growth of *B. vespertilionis* NBRC 113380^{T} (top panel) and *B. aerophilum* JCM 30941^{T} (bottom panel) in medium supplemented with or without 1% gum arabic AG. The samples were taken at the indicated time points for measuring OD_{600} values. Line plots with whiskers represent the mean ± SD of three independent experiments. (**C**) TLC analysis of reaction products following incubation of gum arabic AG with cell suspensions of *B. vespertilionis* NBRC 113380^{T} or *B. aerophilum* JCM 30941^{T}, or with purified recombinant *Ba*Endo/Exo-β1,3-Gal-ase. S5-GA was used as a standard sugar.

Supplementary References

1. Kozakai T, Nakajima A, Miyazawa K et al. An improved temperature-sensitive shuttle vector system for scarless gene deletion in human-gut-associated *Bifidobacterium* species. *iScience* 2024;**27**. doi:10.1016/j.isci.2024.111080

2. Malukiewicz J, D'arc M, Dias CA et al. Bifidobacteria define gut microbiome profiles of golden lion tamarin (*Leontopithecus rosalia*) and marmoset (*Callithrix* sp.) metagenomic shotgun pools. *Sci Rep* 2023;**13**:15679. doi:10.1038/s41598-023-42059-4

3. Viborg AH, Katayama T, Hachem MA et al. Distinct substrate specificities of three glycoside hydrolase family 42 β-galactosidases from *Bifidobacterium longum* subsp. *infantis* ATCC 15697. *Glycobiology* 2014;**24**:208-216. doi:10.1093/glycob/cwt104

4. Cartmell A, Muñoz-Muñoz J, Briggs JA et al. A surface endogalactanase in *Bacteroides thetaiotaomicron* confers keystone status for arabinogalactan degradation. *Nat Microbiol* 2018;**3**:1314-1326. doi:10.1038/s41564-018-0258-8

5. Ling NX, Lee J, Ellis M et al. An exo-β-(1→3)-D-galactanase from *Streptomyces* sp. provides insights into type II arabinogalactan structure. *Carbohydr Res* 2012;**352**:70-81. doi:10.1016/j.carres.2012.02.033

6. Sasaki Y, Horigome A, Odamaki T et al. Novel 3-*O*-α-D-galactosyl-α-L-arabinofuranosidase for the assimilation of gum arabic arabinogalactan protein in *Bifidobacterium longum* subsp. *longum*. *Appl Environ Microbiol* 2021;**87**:e02690-20. doi:10.1128/aem.02690-20

7. Sasaki Y, Togo N, Kitahara K et al. Characterization of a GH36 β-L-arabinopyranosidase in *Bifidobacterium adolescentis*. *J Appl Glycosci* 2018;**65**:23-30. doi:10.5458/jag.jag.JAG-2018_001

8. McKee LS and Brumer H. Growth of *Chitinophaga pinensis* on plant cell wall glycans and characterisation of a glycoside hydrolase family 27 β-L-arabinopyranosidase implicated in arabinogalactan utilisation. *PLoS One* 2015;**10**:e0139932. doi:10.1371/journal.pone.0139932

9. Sasaki Y, Komeno M, Ishiwata A et al. Mechanism of cooperative degradation of gum arabic arabinogalactan protein by *Bifidobacterium longum* surface enzymes. *Appl Environ Microbiol* 2022;**88**:e02187-21. doi:10.1128/aem.02187-21

10. Ichinose H, Fujimoto Z, and Kaneko S. Characterization of an α-L-rhamnosidase from *Streptomyces avermitilis*. *Biosci Biotechnol Biochem* 2013;**77**:213-216. doi:10.1271/bbb.120735

11. Sasaki Y, Uchimura Y, Kitahara K et al. Characterization of a GH36 α-D-galactosidase associated with assimilation of gum arabic in *Bifidobacterium longum* subsp. *longum* JCM7052. *J Appl Glycosci* 2021;**68**:47-52. doi:10.5458/jag.jag.JAG-2021_0004

12. Theilmann MC, Fredslund F, Svensson B et al. Substrate preference of an ABC importer corresponds to selective growth on β-(1,6)-galactosides in *Bifidobacterium animalis* subsp. *lactis*. *J Biol Chem* 2019;**294**:11701-11711. doi:10.1074/jbc.RA119.008843

13. Sá-Nogueira I and Ramos SS. Cloning, functional analysis, and transcriptional regulation of the *Bacillus subtilis araE* gene involved in L-arabinose utilization. *J Bacteriol* 1997;**179**:7705-7711. doi:10.1128/jb.179.24.7705-7711.1997

14. Ichinose H, Kuno A, Kotake T et al. Characterization of an exo-β-1,3-galactanase from *Clostridium*

thermocellum. *Appl Environ Microbiol* 2006;**72**:3515-3523. doi:10.1128/AEM.72.5.3515-3523.2006
15. Fujita K, Sakaguchi T, Sakamoto A et al. *Bifidobacterium longum* subsp. *longum* exo-β-1,3-galactanase, an enzyme for the degradation of type II arabinogalactan. *Appl Environ Microbiol* 2014;**80**:4577-4584. doi:10.1128/aem.00802-14
16. Ichinose H, Kotake T, Tsumuraya Y et al. Characterization of an exo-β-1,3-D-galactanase from *Streptomyces avermitilis* NBRC14893 acting on arabinogalactan-proteins. *Biosci Biotechnol Biochem* 2006;**70**:2745-2750. doi:10.1271/bbb.60365
17. Okawa M, Fukamachi K, Tanaka H et al. Identification of an exo-β-1,3-D-galactanase from *Fusarium oxysporum* and the synergistic effect with related enzymes on degradation of type II arabinogalactan. *Appl Microbiol Biotechnol* 2013;**97**:9685-9694. doi:10.1007/s00253-013-4759-3
18. Kotake T, Kitazawa K, Takata R et al. Molecular cloning and expression in *Pichia pastoris* of a *Irpex lacteus* exo-β-(1→3)-galactanase gene. *Biosci Biotechnol Biochem* 2009;**73**:2303-2309. doi:10.1271/bbb.90433
19. Ichinose H, Yoshida M, Kotake T et al. An exo-β-1,3-galactanase having a novel β-1,3-galactan-binding module from *Phanerochaete chrysosporium*. *J Biol Chem* 2005;**280**:25820-25829. doi:10.1074/jbc.M501024200
20. Sakamoto T, Tanaka H, Nishimura Y et al. Characterization of an exo-β-1,3-D-galactanase from *Sphingomonas* sp. 24T and its application to structural analysis of larch wood arabinogalactan. *Appl Microbiol Biotechnol* 2011;**90**:1701-1710. doi:10.1007/s00253-011-3219-1
21. Paysan-Lafosse T, Blum M, Chuguransky S et al. InterPro in 2022. *Nucleic Acids Res* 2023;**51**:D418-D427. doi:10.1093/nar/gkac993
22. Waterhouse AM, Procter JB, Martin DM et al. Jalview Version 2—a multiple sequence alignment editor and analysis workbench. *Bioinformatics* 2009;**25**:1189-1191. doi:10.1093/bioinformatics/btp033
23. Fu L, Niu B, Zhu Z et al. CD-HIT: accelerated for clustering the next-generation sequencing data. *Bioinformatics* 2012;**28**:3150-3152. doi:10.1093/bioinformatics/bts565
24. Katoh K and Standley DM. MAFFT multiple sequence alignment software version 7: improvements in performance and usability. *Mol Biol Evol* 2013;**30**:772-780. doi:10.1093/molbev/mst010
25. Minh BQ, Schmidt HA, Chernomor O et al. IQ-TREE 2: new models and efficient methods for phylogenetic inference in the genomic era. *Mol Biol Evol* 2020;**37**:1530-1534. doi:10.1093/molbev/msaa015
26. Letunic I and Bork P. Interactive Tree of Life (iTOL) v6: recent updates to the phylogenetic tree display and annotation tool. *Nucleic Acids Res* 2024;**52**:W78-W82. doi:10.1093/nar/gkae268
27. Zheng J, Ge Q, Yan Y et al. dbCAN3: automated carbohydrate-active enzyme and substrate annotation. *Nucleic Acids Res* 2023;**51**:W115-W121. doi:10.1093/nar/gkad328
